# Acute aerobic exercise is associated with a biphasic, temporally ordered shift in serum extracellular vesicle microRNA expression in major depressive disorder

**DOI:** 10.3389/fmolb.2026.1910088

**Published:** 2026-09-15

**Authors:** Beatriz B. Elliott, Viraaj Vishnu Prasad, Alicia Taylor, Ian J. Shofner, Karl Kerns, Jacob Meyer, Elizabeth M. McNeill

**Affiliations:** 1 Department of Food Science and Human Nutrition and Program in Neuroscience, Iowa State University, Ames, IA, United States; 2 Department of Food Science and Human Nutrition and Program in Molecular, Cellular, and Developmental Biology, Iowa State University, Ames, IA, United States; 3 Department of Animal Science, Iowa State University, Ames, IA, United States; 4 Department of Kinesiology, University of Wisconsin, Madison, WI, United States

**Keywords:** acute aerobic exercise, extracellular vesicles, major depressive disorder, microRNA, neuroplasticity, synaptic plasticity

## Abstract

**Introduction:**

Acute aerobic exercise (AAE) is known to reduce symptoms of Major Depressive Disorder (MDD), yet the underlying biological mechanisms remain poorly understood. Here, we profiled microRNA (miRNA) expression within the serum extracellular vesicle (EV) enriched fraction (hereafter 'EV-enriched miRNA') of individuals diagnosed with MDD at multiple timepoints: before, during (mid), immediately after, and 75 min following a single 30-min session of moderate aerobic exercise. By characterizing the temporal dynamics of EV-enriched miRNA responses to AAE, our objective was to uncover molecular changes that may mediate exercise-induced molecular adaptations relevant to short- or long-term antidepressant effects of exercise and to identify candidate EV-enriched miRNAs for future mechanistic investigation of MDD.

**Methods:**

An EV-enriched serum fraction was isolated from a minimum of 28 individuals diagnosed with MDD. MicroRNA profiles were then assessed *via* RNA deep sequencing and analyzed with using DESeq2.

**Results:**

We identified time-dependent, significant (false discovery rate [FDR] ≤ 0.05) changes in EV-enriched microRNAs across AAE, with 15 differentially expressed at mid-AAE, 33 immediately post-AAE, and four at 75 min post-AAE. The response was biphasic and directional: miRNAs were predominantly downregulated during and immediately after exercise (13 of 15 down at mid-AAE; all 33 down post-AAE) and exclusively upregulated during recovery (all four up at 75 min post-AAE). hsa-miR-12523p was the only microRNA differentially expressed across all three postexercise timepoints, showing consistent downregulation. Functional enrichment of predicted targets of significantly differentially expressed miRNAs revealed a temporally ordered shift from RNA processing and stability at mid-AAE, to dendrite, synapse, and axon organization immediately post-AAE, to synaptic transmission and neuronal development at 75 min post-AAE.

**Discussion:**

AAE is associated with time-dependent, directional changes in EV-enriched microRNAs in MDD, whose predicted targets converge on synaptic plasticity pathways implicated in the disorder. Together, this suggests microRNAs may contribute to the beneficial short- and potentially long-term effects of aerobic exercise in individuals with MDD and provides specific targets for future mechanistic investigation.

## 1 Introduction

Major Depressive Disorder, or MDD, is one of the most common psychiatric disorders in the United States, affecting around 20% of adults in their lifetime, and is characterized by persistent depressed mood, anhedonia, and disturbances in sleep, appetite, and concentration (American Psychiatric Association, 2022; Hasin et al., 2018; National Institute of Mental Health, 2023). In recent years, exercise’s acute antidepressant effects have been evaluated as a potential behavioral tool for managing and treating MDD. Acute aerobic exercise (AAE) has consistently been shown to alleviate MDD symptoms, particularly those related to core MDD symptoms, including depressed mood and state anhedonia (Ligeza et al., 2023; Niedermeier et al., 2021; Torelly et al., 2022). Recently, Meyer et al. found that a single 30-min AAE session resulted in significant improvements in depressed mood and state anhedonia in individuals with MDD, with these effects persisting for at least 75 min post-exercise (Meyer et al., 2022). However, the molecular mechanisms underlying these symptom improvements remain unclear.

microRNAs (miRNAs) are small, non-coding RNAs that regulate gene expression at the post-transcriptional level (Shang et al., 2023) and have been implicated in numerous biological processes and pathologies (Li et al., 2021). Accumulating evidence shows that miRNAs are dysregulated in individuals with MDD (Hu et al., 2024; Prodan-Bărbulescu et al., 2024; Wei et al., 2020), and separately, that both acute and chronic aerobic exercise alter circulating miRNA expression (D'Souza et al., 2018; Eyileten et al., 2022; Huang et al., 2024; Li et al., 2020; Maggio et al., 2023; Mayr et al., 2024; Zhou et al., 2020). Together, these findings position miRNAs as a plausible molecular link between AAE and its antidepressant effects.

A growing body of research highlights the role of extracellular vesicles (EVs) in mediating intercellular communication. EVs are lipid bilayer-enclosed nanoparticles that carry cargo, including miRNAs, and can cross the blood-brain barrier (Banks et al., 2020; Kumar et al., 2024), enabling systemic transport of miRNAs (Iannotta et al., 2024). As such, EVs carrying miRNA cargo represent a plausible, though not yet directly tested, link between MDD pathology and the body’s molecular response to exercise.

Despite this evidence, no study has directly examined how serum EV-enriched miRNAs change over time in response to AAE in individuals with MDD. In this study, we address this gap by characterizing the temporal dynamics of EV-enriched miRNA expression in individuals with MDD at four timepoints; pre-exercise, mid-exercise, immediately post-exercise, and 75 min post-exercise, using samples from the Meyer et al. study in which a single AAE session improved depressed mood and state anhedonia up to 75 min post-AAE (Meyer et al., 2022). We hypothesize that AAE will induce time-dependent changes in EV-enriched miRNA expression, with predicted targets of these miRNAs enriched for neuronal pathways relevant to MDD, and that distinct, persistent regulation patterns will emerge that reflect both immediate and delayed (75 min post-AAE) molecular adaptations to exercise, offering candidate molecular targets for future mechanistic investigation.

## 2 Materials and methods

### 2.1 Subjects

This study is a secondary analysis of serum samples originally collected as part of the *Exercise and Brain Health Study* conducted by Meyer and colleagues (Meyer et al., 2022) (see for full study protocol and primary outcomes). Here, we focus solely on the subset of methods relevant to the present analysis of EV-enriched miRNAs in adults with MDD.

Participants in the original study were 30 adults (21 female; mean age = 30.4 ± 9.1 years) with a current diagnosis of MDD, confirmed using the Mini International Neuropsychiatric Interview 7.0.2 (Sheehan et al., 1998). Inclusion criteria required individuals to be between 20 and 60 years of age, able to complete 30 min of moderate-intensity physical activity, and experiencing a major depressive episode (MDE) characterized by five or more symptoms (including at least one core symptom) with associated distress in the preceding 2 weeks (determined by a structured clinical interview). Additional inclusion/exclusion criteria and full participant demographics are detailed in Meyer et al. Blood sample collection for the *Exercise and Brain Health Study* was approved by the Iowa State University Institutional Review Board.

### 2.2 Study design

Participants underwent a 30-min bout of moderate-intensity aerobic exercise using a recumbent cycle ergometer. During exercise, intensity was guided by a perceived exertion rating (RPE) of 13 using Borg’s 6–20 scale (Meyer et al., 2022). After a 3–5-min warm-up, participants self-adjusted the resistance to maintain this exertion level while pedaling at 60–80 RPM. This protocol was previously validated in the same population and associated with moderate physiological intensity (mean respiratory exchange ratio: 0.96; HR: 130.8 bpm) (Meyer et al., 2016). All measurements (pre-, mid-, and post-condition) were taken while seated, and a 75-min post-condition period included quiet rest with non-stimulating magazines without electronic device use.

### 2.3 Blood collection and processing

Blood samples were collected at multiple time points to assess physiological response. Serum was collected in BD Vacutainer Serum Tubes, allowed to clot for 60–90 min, centrifuged for 10 min, before being aliquoted into 1.5 mL microcentrifuge tubes and stored at −80 °C until processing. The time points were pre-, mid-, post-, and 75 min post-AAE, corresponding to the baseline before the intervention, the midpoint of the 30-min session, immediately after the intervention, and 75 min after the intervention, respectively. Blood draws were performed while participants were seated on the ergometer in pre-, mid-, and post-conditions and while seated in a chair at 75 min to maintain consistency across conditions (Figure 1).

**FIGURE 1:**
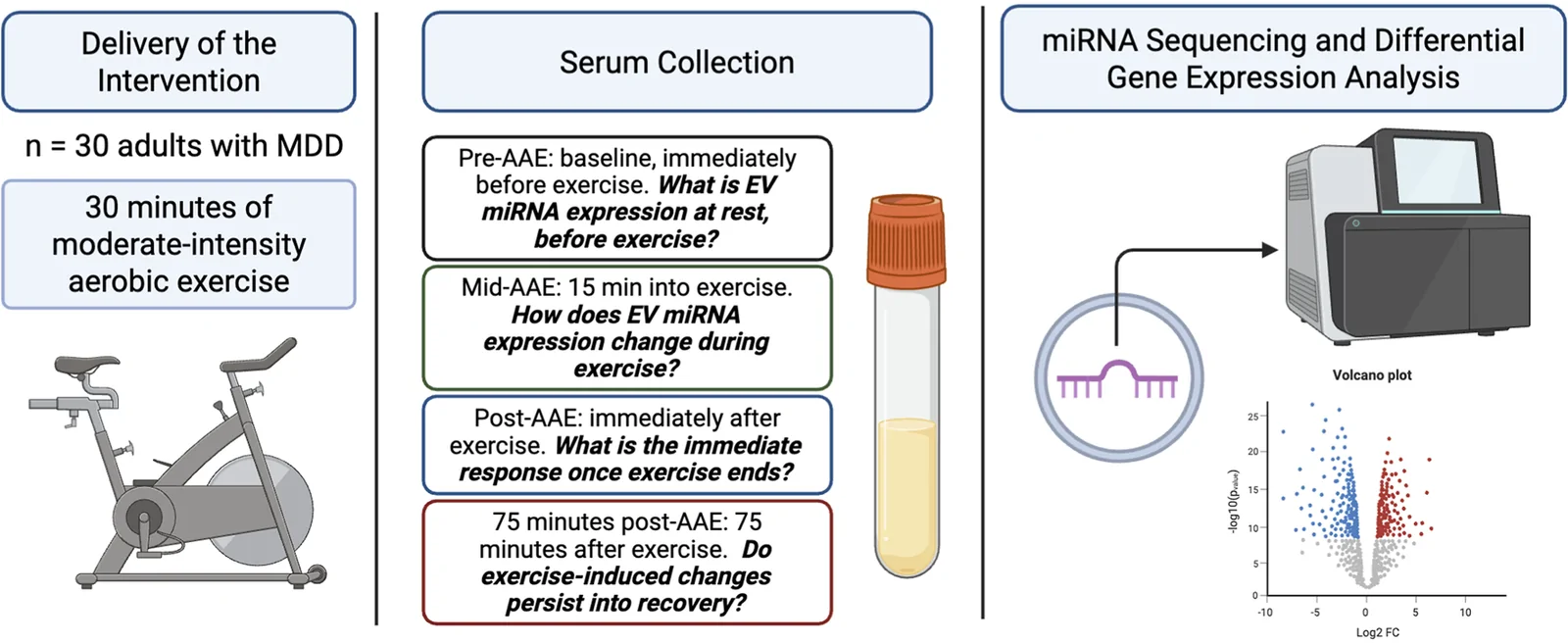
Schematic overview of study design. Individuals diagnosed with Major Depressive Disorder were placed on a recumbent cycle ergometer where they underwent 30 min of moderate-intensity aerobic exercise (AAE). Next, blood was collected and processed into serum at four timepoints: pre-AAE, before beginning a 30-min bout of AAE; mid-AAE, in the middle of the 30-min bout; post-AAE, immediately after the 30-min bout; and 75 min post-AAE, or 75 min after the 30-min bout of AAE on the recumbent cycle ergometer. EV RNA was isolated from collected serum, then miRNA libraries were sequenced on an Illumina NovaSeq 6000 platform to analyze differential gene expression. Colors used to represent each timepoint (pre-AAE = black, mid-AAE = green, post-AAE = blue, 75 min post-AAE = orange) are used throughout the manuscript. Created in BioRender. Pereira, B. (2026) https://BioRender.com/xox0r01.

### 2.4 EV isolation and characterization

#### 2.4.1 EV isolation

Serum samples were filtered through a 0.8 µm membrane (sterile, Sartorius, Cat. No. 17594-K, Göttingen, Germany) to remove larger cellular debris. According to manufacturer instructions, EVs were isolated using the commercially available exoEasyMaxi Kit (Qiagen, Cat. No. 76064, Hilden, Germany) from 200 µL of filtered serum. The serum EV-enriched fraction was eluted in 400 µL of the provided elution buffer and stored at −80 until further RNA extractions. A 2 µL aliquot was set aside for four randomly selected samples that underwent Transmission Electron Microscopy (TEM) at the Roy J. Carver High-Resolution Microscopy Facility. The presence of EVs was validated using TEM (See Supplementary Materials).

#### 2.4.2 Transmission Electron Microscopy

Isolated EV fraction (1 µL) from human serum was sterilized in 50% stock concentrated glutaraldehyde, then applied to carbon-coated copper grids and adsorbed for 1 min. Vesicles were negatively stained with 0.5% uranyl acetate for 1 min, blotted, and air-dried. Images were acquired on a 200 kV JEOL 2100 scanning/transmission electron microscope equipped with a Gatan CCD camera at the Roy J. Carver High Resolution Microscopy Facility (Iowa State University).

#### 2.4.3 Vesicle flow cytometry

EV size, concentration, and surface cargo were measured by single-vesicle flow cytometry (vFC) using the vFC Assay Kit (Cellarcus Biosciences, San Diego, CA) on an ImageStream®X Mark II. Detection was triggered on vFRed fluorescence (blue-excited, B6-H), with the threshold set to yield 10–15 events/s in a buffer-only sample.

Pre-stain dilutions were derived from particle counts obtained by vFC Protocol 1. Samples (n = 4) (5 µL) were stained in 50 µL vFC Staining Buffer containing vFRed membrane stain, with or without PE-conjugated anti-human tetraspanin antibody (CD9/CD63/CD81), for 60 min at room temperature in the dark. Reactions were then diluted 1:30 (5 µL into 145 µL Staining Buffer) and acquired for 5 min at fixed acquisition. Controls comprised buffer alone, buffer plus all reagents, Lipo100™ synthetic liposomes (antigen-negative), and the Cellarcus EV Reference Preparation (positive control).

Tetraspanin-positive events (CD9/CD63/CD81-PE) were identified using gates set relative to the Lipo100™ antigen-negative control. Vesicle diameter was calibrated from vFRed fluorescence intensity against the Lipo100™ liposome standard in FCS Express (*De Novo* Software).

### 2.5 RNA extraction and sequencing

For total RNA extraction, EV-enriched fractions were thawed on ice and then processed using the miRNeasy Serum/Plasma kit (Qiagen, Cat. No. 217184, Hilden, Germany) according to manufacturer instructions. miRNA concentrations were quantified using the Qubit™ microRNA Assay Kit (Thermo Fisher Scientific, Cat. No. Q32880, Waltham, MA, USA) according to the manufacturer’s instructions. Small RNAs' quality and size distribution were assessed using the Agilent Small RNA Kit (Agilent Technologies, Cat. No. 5067-1548, Santa Clara, CA, USA) on the Agilent 2100 Bioanalyzer. RNA was purified using the RNA Clean & Concentrator™ kit (Zymo Research, Cat. No. R1015, Irvine, CA, USA) according to the manufacturer’s instructions. Total RNA samples were stored at −80 °C until further processing.

Small RNA libraries were made with the Lexogen Small RNA-Seq Library Prep Kit using 6 μL of sample per reaction. Alterations to the protocol due to low serum EV RNA concentration include a 1:5 dilution of adaptors A3, A5, and primer RTP, along with 22 qPCR cycles. Library concentration and size distribution was analyzed using Qubit™ dsDNA HS Assay Kit (Thermo Fisher Scientific, Cat. Nos. Q32851, Q32854) and the Agilent 2100 Bioanalyzer HS DNA assay (Agilent Technologies, Santa Clara, CA, USA). Library preparation was unsuccessful for ten samples with low RNA concentration, with a final participant count of 30 pre-, 29 mid-, 28 post-, and 28 75-min post-AAE. Sequencing was performed on an Illumina NovaSeq 6000 platform with single-end, 100-cycle reads on an S4 cell, with 30–50 estimated reads per sample. Samples were indexed and multiplexed into four pools; each loaded into four lanes. Libraries were sequenced at the DNA Facility at Iowa State University.

### 2.6 RNA data processing and quality control

Samples were received as fastq files and processed on a Unix-based operating system. FastQC^1^ v0.12.1 was used for quality control of read length distribution, over-represented sequences, and base quality. Adaptor removal was conducted with BBMap^2^ v38.18 with 3′ end trimming, allowing up to two mismatches, removing the small RNA 3′ adaptor sequence “TGGAATTCTCGGGTGCCAAGGAACTCCAGTCAC”. Trimmed reads were aligned to miRBase mature sequences (mature.fa) using Bowtie v1.3.1 (Langmead et al., 2009) in end-to-end alignment mode (-v 1), allowing up to one mismatch across the full read length. Reads aligning to more than one reference sequence within this tolerance were excluded from analysis (-m 1) to prevent ambiguous assignment across miRNA families. As isomiRs were not resolved as distinct entities, reads meeting these criteria were collapsed into their corresponding canonical mature miRNA counts. A total count file was created using Samtools (Danecek et al., 2021) v1.21. An Isolation Forest model was applied to normalized small RNA-seq counts using the isotree (Liu et al., 2008) R package v 0.6.1.4 (ntrees = 100). Samples with outlier scores above the 95th percentile were considered potential outliers and further evaluated. Principal component analysis plot inspection, along with Cook’s distance, allowed for further evaluation of outliers. No outliers were detected.

### 2.7 Statistical analysis

Differential expression was assessed in R (R Core Team, 2024) (v4.4.0) using the DESeq2 (Love et al., 2014) package (v1.44.0), which models count data based on the negative binomial distribution. Raw counts were normalized using DESeq2’s default size factor estimation using estimateSizeFactors(), computed from the complete, unfiltered count matrix. Low-abundance miRNAs were excluded by retaining only those with at least 10 DESeq2-normalized counts in at least three samples. We applied this threshold to DESeq2-normalized counts as an additional, conservative step to reduce the influence of low-abundance, high-variance miRNAs on downstream dispersion estimation (Love et al., 2014). The design formula was specified as ∼ ID + Treatment, treating both variables as factors to account for within-subject variability. Model convergence was verified, and genes failing to converge (2%) were excluded. Wald tests were used to evaluate significance, and p-values were adjusted for multiple comparisons using the Benjamini–Hochberg procedure. Pairwise contrasts were specified using the contrast argument in the results() function to compare each post-exercise time point against baseline (pre-AAE, the reference level for the ‘Treatment’ factor when performing pairwise comparisons). This within-subject design enabled control for inter-individual variability and enhanced sensitivity to exercise-induced transcriptional changes. miRNAs with adjusted p-values ≤0.05 were considered significantly differentially expressed; those with adjusted p-values ≤0.10 were classified as trending.

Statistical procedures were embedded within bioinformatic workflows for target prediction and pathway analysis. Predicted mRNA targets of differentially expressed miRNAs were identified using DIANA-microT 2023 (Tastsoglou et al., 2023), applying a minimum interaction score threshold of 0.8. Redundant and unannotated genes were excluded prior to downstream analyses. To assess temporal overlap in target gene sets, shared targets were visualized with a Venn diagram and pairwise overlap coefficients were computed between mid-, post-, and 75 min post-AAE conditions; the overlap coefficient (shared targets divided by the size of the smaller set) was used because it is less sensitive to differences in set size than the Jaccard index. These coefficients were visualized as a heatmap to illustrate the degree of overlap across time points. Functional enrichment of predicted targets was conducted using PANTHER (Thomas et al., 2022) with reference to the GO Biological Processes Complete dataset, using the whole human genome as the reference background. Processes with Bonferroni-adjusted p-values <0.05 were considered statistically significant. Among significant processes, the leading terms were ranked by fold enrichment to emphasize biological effect size, and bubble plots were generated in R to visualize the most enriched terms at each time point.

All code used for count processing, differential expression modeling, and visualization is publicly available *via* GitHub repository, https://github.com/bpereira2022/MDD.git.

## 3 Results

### 3.1 Serum extracellular vesicle characterization

Transmission electron microscopy confirmed the presence of vesicular structures the isolated EV fraction. Negative staining revealed round to cup-shaped vesicular structures, with defined membranes (Figure 2A). Vesicles appeared intact, and the observed morphology and distribution of size are consistent with previously published literature with the use of the exoEasyMaxi Kit (Qiagen, Cat. No. 76064, Hilden, Germany).

**FIGURE 2:**
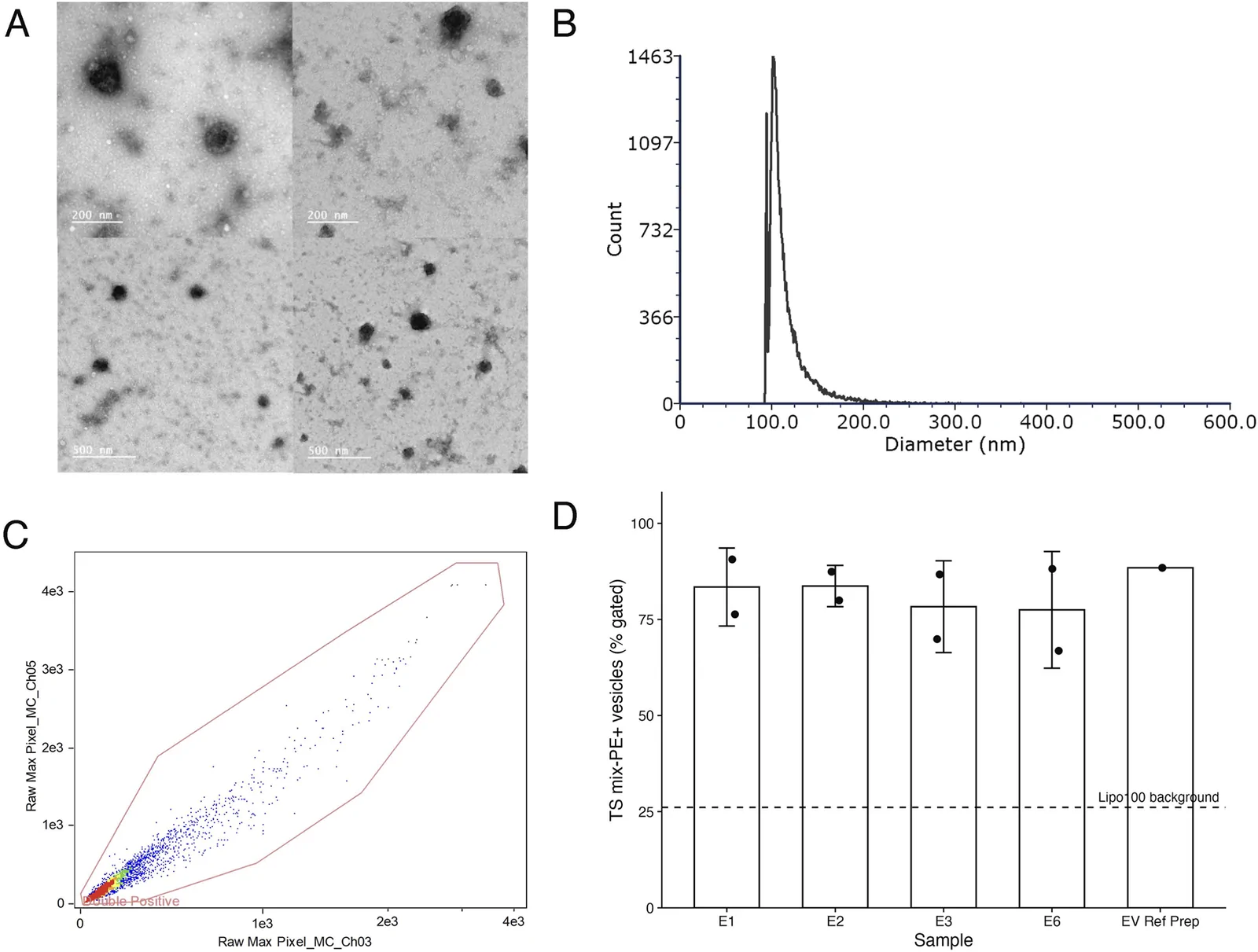
Characterization of serum-derived extracellular vesicles (EVs). **(A)** Representative transmission electron micrographs of isolated EVs, showing the expected cup-shaped/spherical vesicle morphology at two magnifications (scale bars, 200 nm and 500 nm) (n = 1). **(B)** Representative single-vesicle diameter histogram from ImageStream analysis of vFRed-labeled particles (sample E3, replicate 2), showing the size distribution (0–600 nm) used to calculate the median diameter, geometric mean, and CV reported in Supplementary Table 1. **(C)** Representative ImageStream dot plot showing dual-fluorescence gating of vesicle events by tetraspanin-mix-PE (Channel 3) and vFRed (Channel 5) fluorescence intensity (arbitrary units, AU); the gated region (red outline) denotes double-positive events used to define *bona fide* EVs. **(D)** Percentage of double-positive (TS mix-PE^+^) vesicle events gated per sample, shown as mean ± SD across replicates for four biological samples collected at different timepoints (E1, E2, E3, E6; n = 2 replicates each) alongside an EV reference preparation (n = 1), with individual replicate values overlaid as points. The dashed line indicates the Lipo100 liposome background threshold, confirming tetraspanin-labeling specificity above background.

Vesicle size was measured by vFC using vFRed membrane fluorescence calibrated against the Lipo100™ synthetic vesicle standard. A median of 27,101 vesicle events (mean 25,850 ± 7,027) were captured within the Vesicles gate per replicate, with good reproducibility between technical replicates (mean %CV = 8.5%); event counts varied more broadly across sample preparations (%CV = 27.2%), consistent with expected biological variability in EV yield. All samples displayed narrow, right-skewed size distributions with median diameters of 103–107 nm (geometric mean 106–112 nm; Supplementary Table 1). A representative size distribution is provided in Figure 2B.

Representative bivariate gating of a double-positive vesicle population (PE-tetraspanin vs. vFRed) is shown in Figure 2C. Across all EV preparations, tetraspanin surface marker expression (CD9/CD63/CD81-PE) was elevated relative to the Lipo100 antigen-negative control (26.1% double-positive; Figure 2D). E1 and E2 showed the highest double-positive signal (∼83%), E3 and E6 were slightly lower (∼77–78%), and the EV reference preparation showed the greatest overall positivity (88%) (Figure 2D). Together, these results demonstrate specific tetraspanin antibody binding to isolated EV populations above assay background.

### 3.2 miRNA sequencing quality control assessment

Quality control was performed on pre- and post-trimming miRNA-seq samples using FastQC and MultiQC. Prior to trimming, the median read count per sample was 53.7 million, with a fixed read length of 50 bp. Following adapter trimming, the median read count per sample was 31.1 million (range: 25,506–353 million), reflecting variability in RNA/EV yield from serum samples collected under observational biological conditions. This read-depth heterogeneity is recognized as a feature of small RNA-Seq from clinically collected human biofluids and could reduce sensitivity for low-abundance miRNAs in the lowest-depth libraries. Two low-count libraries underwent sensitivity analysis using dispersion trend and Cook’s distance diagnostics, which did not indicate that these libraries disproportionately influenced differential expression results. Read length had a median of 26 bp (range: 19–50 bp), consistent with mature miRNAs. Reads at the upper end of this range likely reflect a small subset in which the 3′adapter was not detected during trimming and were therefore retained at their original sequenced length, as no minimum-length filter was applied. As these longer reads cannot align end-to-end to the short (∼18–24 nt) mature miRNA reference sequences used in our alignment step, they did not contribute to final miRNA counts. All samples passed the basic_statistics, per_base_sequence_quality, per_sequence_quality_scores, per_base_n_content, and adapter_content modules. After variance-stabilizing transformation, the range of library sizes was more uniform, as expected (Supplementary Figure 1a). Per-base sequence quality remained high, with no poor-quality reads detected, and the average GC content was 49%. Most samples passed all metrics for adapter content, confirming the effectiveness of the trimming process. Overall, these post-trimming QC metrics indicate a substantial improvement in base-level read quality.

Isotree analysis, Cook’s distance, and PCA collectively revealed no obvious outliers among the samples, indicating consistent data quality across the dataset (Supplementary Figures 1b, c).

Reads were successfully aligned to mature human miRNAs in miRBase v22, with an average alignment percentage rate of 2.02%. This is consistent with published small RNA-seq studies of serum-derived EVs, where non-miRNA small RNA species such as rRNA and tRNA fragments constitute the majority of reads (Buschmann et al., 2018).

Our data successfully fit into a negative binomial model (Supplementary Figure 1d). After filtering out low-abundance miRNAs (defined as those with fewer than ten counts in at least three samples), 2,609 unique mature miRNAs were identified, with a median of 560,077 counts per sample. Among these, the five most highly expressed miRNAs in serum EVs from participants with MDD were hsa-miR-6500-3p, hsa-miR-4538, hsa-miR-4539, hsa-miR-590-5p, and hsa-miR-4728-5p (Supplementary Figure 1e).

As a *post hoc* assessment of hemolysis, we computed a hemolysis index (log2 miR-451a:miR-23a-3p ratio) for all samples. Indices ranged widely across individuals (range: −6.49–12.24), with several samples exceeding the conventional hemolysis threshold (log2 ratio >2.81) at each timepoint. A Friedman test revealed no significant difference in hemolysis index across timepoints (p = 0.472), indicating that although individual-level variation was present, hemolysis did not systematically differ as a function of the exercise intervention. A principal component analysis colored by hemolysis index revealed no visual clustering (Supplementary Figure 2).

We additionally examined five platelet-enriched miRNAs across timepoints as a proxy for platelet contamination, using Friedman tests with Benjamini–Hochberg correction (Supplementary Figure 3). None reached significance after correction (all p-adj ≥0.167). miR-21-5p and miR-92a-1-5p, both independently identified as differentially expressed in the primary analysis, showed visibly greater timepoint-associated variability, and miR-21-5p reached nominal significance prior to correction (p = 0.049); miR-223-3p, miR-126-3p, and miR-191-5p remained relatively stable. These results suggest platelet contamination is unlikely to account for the temporal miRNA patterns observed in the primary analysis.

### 3.3 Differential gene expression analysis

Principal component analysis of normalized miRNA expression revealed substantial overlap among samples collected at mid-, post-, and 75 min AAE with pre-AAE samples (Supplementary Figure 4). This suggests that, at the global level, AAE does not induce large-scale shifts in the miRNA expression landscape.

Despite the lack of global clustering, pairwise differential expression analysis identified multiple miRNAs whose abundance was significantly altered following AAE. Compared to pre-exercise, 15 miRNAs were significantly differentially expressed at mid-AAE, 33 miRNAs at post-AAE, and four miRNAs at 75 min post-AAE (FDR ≤0.05; Figures 3A–C). The response was biphasic and directionally consistent: at mid-AAE, 13 miRNAs were downregulated and two upregulated; immediately post-AAE, all 33 differentially expressed miRNAs were downregulated; and at 75 min post-AAE, all four differentially expressed miRNAs were upregulated. Thus, EV-enriched miRNAs were predominantly suppressed during and immediately after exercise and exclusively elevated during early recovery. All differentially expressed miRNAs, including their log2 fold change, base mean, and adjusted p-values, are presented in Table 1. Significance was defined as FDR ≤0.05; miRNAs with FDR ≤0.10 were considered as trending and are also reported in Table 1.

**FIGURE 3:**
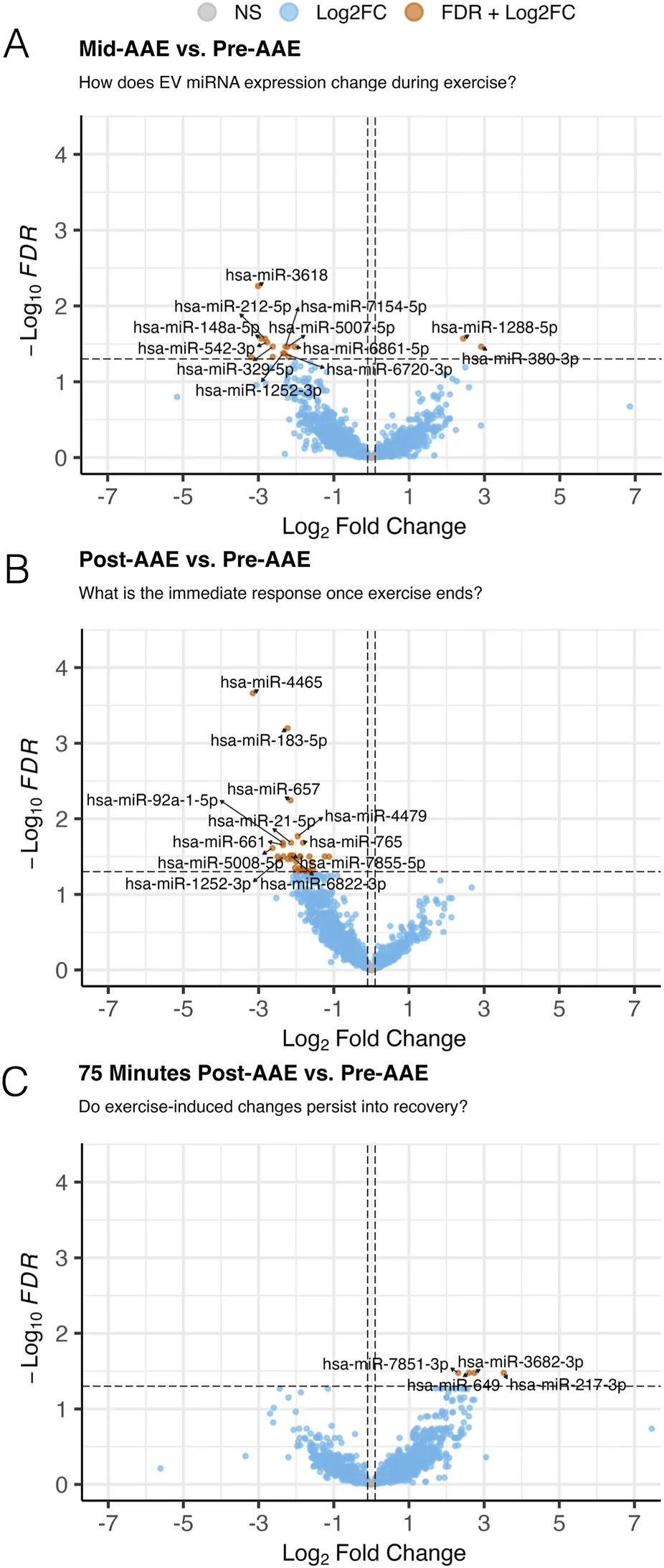
Differential expression of serum EV-enriched miRNAs across acute aerobic exercise. Volcano plots displaying the log2 fold change (x-axis) and -log10(FDR) (y-axis) of EV-enriched miRNAs at **(A)** mid-AAE, **(B)** post-AAE, and **(C)** 75 min post-AAE, each relative to pre-AAE. The horizontal dashed line marks the FDR significance threshold (FDR ≤0.05), and the vertical dashed lines mark the log2 fold change cut-off. miRNAs meeting the fold change threshold are shown in blue, and those meeting both the fold change and FDR thresholds in orange; non-significant miRNAs are grey.

**TABLE 1:** Differentially expressed miRNAs across acute aerobic exercise timepoints.

miRNA	baseMean	log2FoldChange	padj
Mid-AAE vs. Pre-AAE			
hsa-miR-3618	22.6	−3.01	0.0055
hsa-miR-1288-5p	17.8	2.44	0.027
hsa-miR-148a-5p	11.8	−2.92	0.027
hsa-miR-212-5p	18.2	−2.83	0.027
hsa-miR-542-3p	17.9	−2.77	0.03
hsa-miR-329-5p	15.6	−2.62	0.034
hsa-miR-380-3p	5.9	2.91	0.034
hsa-miR-5007-5p	81.4	−2.22	0.034
hsa-miR-6861-5p	101.2	−2.04	0.034
hsa-miR-7154-5p	17.4	−2.28	0.034
hsa-miR-1252-3p	44.3	−2.34	0.042
hsa-miR-6720-3p	14	−2.3	0.043
hsa-miR-137-3p	9.2	−3.19	0.047
hsa-miR-4496	6.8	−2.62	0.047
hsa-miR-6780a-3p	15.4	−2.2	0.047
hsa-miR-3140-3p	18.9	−2.02	0.056
hsa-miR-6887-5p	74.7	−1.62	0.056
hsa-miR-3155a	34.8	−2.09	0.062
hsa-miR-6783-5p	59.1	−1.88	0.063
hsa-miR-3182	15	2.49	0.065
hsa-miR-3605-3p	19.3	−1.5	0.065
hsa-miR-627-3p	6.3	−2.66	0.065
hsa-miR-1267	23.8	−2.03	0.067
hsa-miR-520d-5p	6.4	−2.68	0.067
hsa-miR-657	72	−1.6	0.07
hsa-miR-6499-5p	268.2	−1.19	0.074
hsa-miR-6814-5p	8.9	−2.18	0.074
hsa-miR-450a-2-3p	19.1	−2.22	0.077
hsa-miR-4681	31.1	1.96	0.08
hsa-miR-10399-5p	19.7	−2.23	0.09
hsa-miR-1273c	24	−2.04	0.09
hsa-miR-224-5p	23.6	−1.66	0.09
hsa-miR-4763-3p	33.6	−1.78	0.09
hsa-miR-4796-3p	20.8	2.15	0.091
hsa-miR-8080	21.4	−1.94	0.096
hsa-miR-3651	51	−1.37	0.098
hsa-miR-548az-5p	4.9	2.27	0.098
hsa-miR-3650	7.9	−2.25	0.099
hsa-miR-609	46.8	−1.7	0.099
hsa-miR-6845-5p	17.8	−1.75	0.099
hsa-miR-6744-3p	26.3	−1.81	0.1
Post-AAE vs. Pre-AAE			
hsa-miR-4465	15.3	−3.15	2.20E-04
hsa-miR-183-5p	223.9	−2.23	6.30E-04
hsa-miR-657	72	−2.15	0.0057
hsa-miR-4479	80.7	−1.96	0.017
hsa-miR-21-5p	84.4	−2.14	0.021
hsa-miR-765	57.8	−1.82	0.021
hsa-miR-92a-1-5p	29.6	−2.36	0.021
hsa-miR-661	21.8	−2.35	0.022
hsa-miR-5008-5p	46.6	−2.63	0.024
hsa-miR-6822-3p	53.3	−2.15	0.031
hsa-miR-7855-5p	17.9	−2.08	0.031
hsa-miR-1252-3p	44.3	−2.34	0.032
hsa-miR-1268a	52	−1.65	0.032
hsa-miR-130a-5p	21.3	−2.49	0.032
hsa-miR-224-5p	23.6	−1.9	0.032
hsa-miR-4687-3p	5811.1	−1.13	0.032
hsa-miR-4695-5p	71.7	−1.24	0.032
hsa-miR-4746-3p	19.6	−2.14	0.032
hsa-miR-6127	98.7	−1.89	0.032
hsa-miR-3682-5p	46	−2.13	0.034
hsa-miR-4458	40.7	−2.22	0.034
hsa-miR-7157-3p	16.1	−2.44	0.035
hsa-miR-4446-3p	36.4	−1.99	0.037
hsa-miR-4292	260.8	−1.59	0.038
hsa-miR-625-3p	43.7	−2.03	0.045
hsa-miR-6783-5p	59.1	−1.89	0.045
hsa-miR-4523	157.2	−1.55	0.047
hsa-miR-4804-5p	27.5	−1.77	0.047
hsa-miR-6758-5p	24.4	−1.91	0.048
hsa-miR-8056	25.8	−1.78	0.048
hsa-miR-940	95.5	−1.64	0.048
hsa-miR-1911-5p	48.3	−2.01	0.05
hsa-miR-4685-5p	199	−1.73	0.05
hsa-miR-4467	34.5	−1.95	0.052
hsa-miR-1303	38	−2.01	0.054
hsa-miR-6808-5p	103.6	−1.37	0.054
hsa-miR-10526-3p	272.6	−0.9	0.057
hsa-miR-3156-5p	67.2	−1.59	0.057
hsa-miR-3196	90.8	−1.39	0.057
hsa-miR-33b-3p	43.6	−1.73	0.057
hsa-miR-3616-3p	44.4	−2	0.057
hsa-miR-4769-3p	53.8	−1.66	0.057
hsa-miR-596	27.2	−1.86	0.057
hsa-miR-6732-5p	73.9	−1.06	0.057
hsa-miR-6783-3p	37.3	−1.46	0.057
hsa-miR-6831-5p	56.3	−1.16	0.057
hsa-miR-6879-5p	34	−1.37	0.057
hsa-miR-6887-5p	74.7	−1.49	0.057
hsa-miR-196a-1-3p	92.9	−1.67	0.058
hsa-miR-25-3p	49.2	−2.08	0.058
hsa-miR-4758-3p	64.9	−1.59	0.058
hsa-miR-4767	98.3	−1.64	0.058
hsa-miR-6511b-5p	113.7	−1.26	0.058
hsa-miR-652-5p	30.3	−1.78	0.058
hsa-miR-4772-3p	23.5	−1.99	0.059
hsa-miR-711	41.5	−1.55	0.06
hsa-miR-6741-5p	205.3	−1.28	0.061
hsa-miR-1295b-3p	35.3	−1.71	0.065
hsa-miR-3928-3p	45.7	1.84	0.065
hsa-miR-4313	104.4	−1.64	0.065
hsa-miR-4758-5p	239.7	−1.25	0.065
hsa-miR-6742-5p	34.3	−1.93	0.065
hsa-miR-6769a-3p	40	−1.47	0.065
hsa-miR-6805-5p	35.7	−1.44	0.065
hsa-miR-1-3p	306.9	−1.99	0.066
hsa-miR-6858-5p	103.1	−1.88	0.067
hsa-miR-1976	23.5	−2.1	0.07
hsa-miR-501-3p	53.1	−1.5	0.071
hsa-miR-6802-5p	147.5	−1.36	0.081
hsa-miR-6873-5p	21.9	−1.88	0.081
hsa-miR-888-5p	13.5	2.67	0.081
hsa-miR-4474-3p	26.3	−1.71	0.085
hsa-miR-3911	32.7	−1.58	0.087
hsa-miR-331-5p	34.9	−1.77	0.092
hsa-miR-4787-3p	108.4	−1.23	0.092
hsa-miR-4488	79.3	−1.3	0.093
hsa-miR-6499-5p	268.2	−1.04	0.093
hsa-miR-6715b-5p	21	−1.98	0.093
hsa-miR-2114-5p	82.8	−1.26	0.095
hsa-miR-4726-3p	68.6	−1.44	0.095
hsa-miR-6889-5p	94.4	−1.13	0.095
hsa-miR-933	18	−1.36	0.095
hsa-miR-6885-5p	52.9	−1.41	0.096
hsa-miR-2861	637.8	−1.08	0.099
hsa-miR-3618	22.6	−1.8	0.099
hsa-miR-3651	51	−1.23	0.099
hsa-miR-3944-3p	62.4	−1.4	0.099
hsa-miR-4256	17.1	−1.67	0.099
hsa-miR-4283	137	−1.4	0.099
hsa-miR-4447	88.8	−1.41	0.099
hsa-miR-520d-3p	15.6	−1.95	0.099
hsa-miR-5586-5p	16.5	−2.09	0.099
hsa-miR-5693	49.9	−1.58	0.099
hsa-miR-642b-3p	33.7	−1.59	0.099
hsa-miR-6511a-5p	115.8	−1.08	0.099
hsa-miR-6752-5p	83.9	−1.2	0.099
hsa-miR-6773-5p	36.5	−1.87	0.099
75 min Post-AAE vs. Pre-AAE			
hsa-miR-217-3p	6.4	3.52	0.034
hsa-miR-3682-3p	17.1	2.74	0.034
hsa-miR-649	18.1	2.59	0.034
hsa-miR-7851-3p	22.2	2.31	0.034
hsa-miR-1246	71.3	2.09	0.054
hsa-miR-143-3p	720.1	1.71	0.054
hsa-miR-148b-3p	60.1	1.86	0.054
hsa-miR-196b-3p	44.6	2.45	0.054
hsa-miR-3675-5p	38.9	−1.16	0.054
hsa-miR-3917	15.7	2.5	0.054
hsa-miR-3921	18.3	2.56	0.054
hsa-miR-4423-3p	30.9	2.23	0.054
hsa-miR-4772-3p	23.5	−2.44	0.054
hsa-miR-5579-3p	182.8	2.36	0.054
hsa-miR-6850-3p	105.3	1.85	0.054
hsa-miR-579-3p	7.6	2.05	0.06
hsa-miR-7158-3p	31	−1.87	0.06
hsa-miR-1252-3p	44.3	−2.2	0.071
hsa-miR-1273h-3p	22.3	2.36	0.071
hsa-miR-3683	11.7	2.47	0.071
hsa-miR-4433b-3p	90.4	2	0.071
hsa-miR-512-5p	20.6	2	0.071
hsa-miR-126-3p	56.5	2.17	0.076
hsa-miR-23b-3p	17.9	2.69	0.076
hsa-miR-655-3p	10	2.74	0.076
hsa-miR-5701	2.5	2.38	0.077
hsa-miR-30e-5p	21.7	2.15	0.079
hsa-let-7c-3p	10.3	2.61	0.097
hsa-miR-520d-5p	6.4	−2.59	0.097
hsa-miR-575	6.2	2.4	0.097

Differential expression analysis was performed using DESeq2 (∼ ID + Treatment design) comparing three post-exercise timepoints (mid-AAE, post-AAE, 75 min post-AAE) to the pre-AAE, baseline. MicroRNAs, were filtered for expression (≥10 normalized counts in ≥3 samples). Columns show miRNA, identifier, baseMean (average normalized count), log_2_FoldChange, and padj (Benjamini–Hochberg adjusted p-value). Entries are ordered by statistical significance within each time point. Negative log_2_FoldChange indicates downregulation; positive indicates upregulation.

Most differentially expressed (DE) miRNAs were unique to individual time points, indicating a temporally dynamic response to AAE (Figure 4A). At a less stringent threshold (FDR ≤0.10), a subset of miRNAs was shared across at least two time points (Figures 4B,C). Notably, hsa-miR-1252-3p was the only miRNA differentially expressed across all three post-exercise time points, and it was downregulated at each with a stable effect size: mid-AAE (log2 fold change = −2.34, FDR = 0.042), post-AAE (log2 fold change = −2.34, FDR = 0.032), and 75 min post-AAE (log2 fold change = −2.20, FDR = 0.071). This makes hsa-miR-1252-3p the most temporally persistent EV-enriched miRNA signal in our dataset.

**FIGURE 4:**
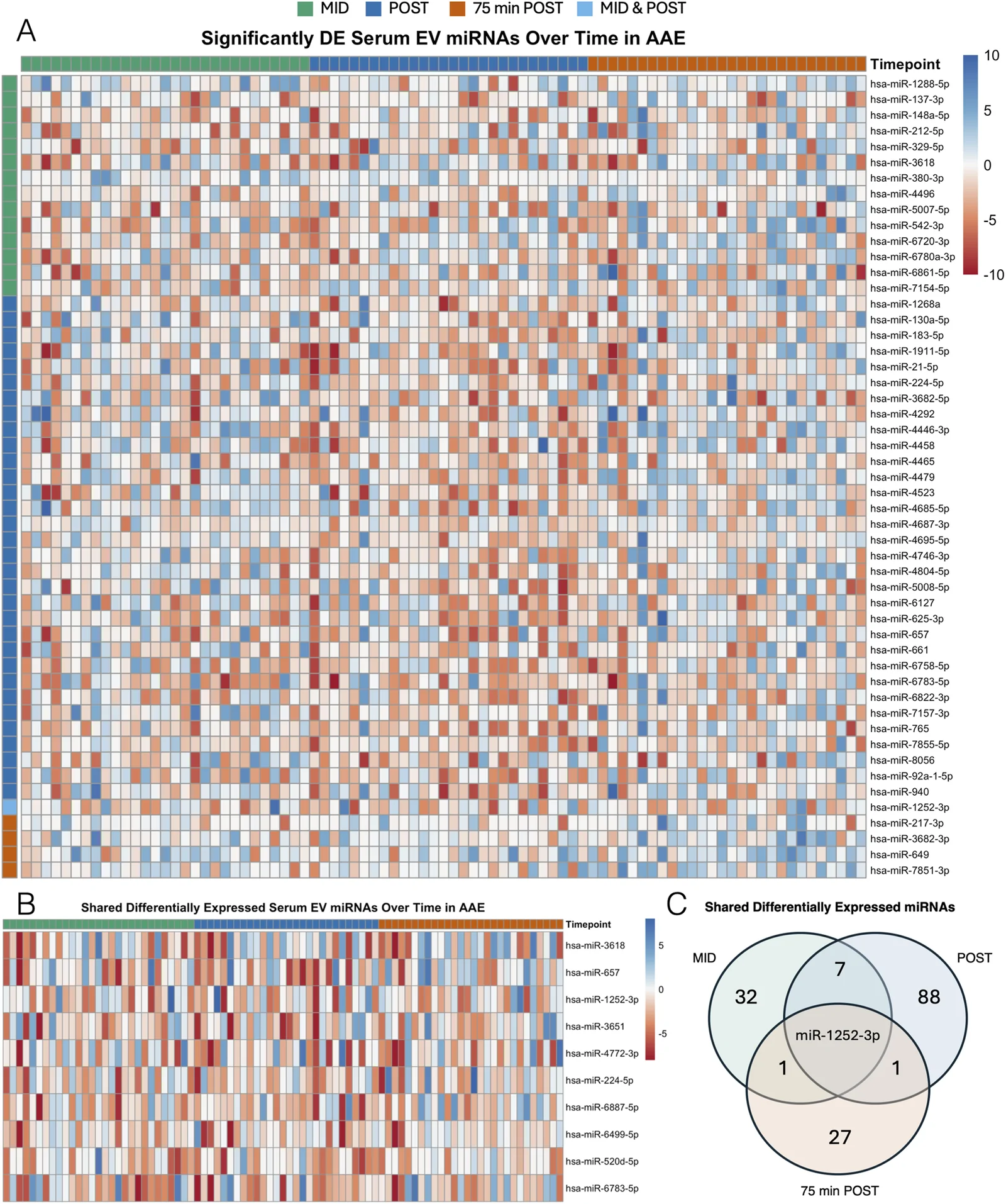
Patterns in differential miRNA expression in acute aerobic exercise over time. **(A)** Heatmap showing log2 fold changes for differentially expressed (DE) miRNAs across mid-, post-, and 75 min post-AAE, relative to pre-AAE. Columns represent individual samples grouped by time point, with rows for significantly differentially expressed miRNAs (FDR ≤0.05). Mid-AAE DE miRNAs are in green, post-AAE in blue, 75 min post-AAE in purple, and miRNAs shared across time points in light blue. **(B)** Heatmap of shared DE miRNAs across at least two time points, considering trending FDR values (FDR ≤0.10) relative to pre-AAE. Upregulation is indicated in blue and downregulation in red. **(C)** Venn diagram of total DE miRNAs (FDR ≤0.10) at each time point and the miRNAs shared across time points; hsa-miR-1252-3p was the only miRNA shared across all three post-exercise time points.

### 3.4 Pathway enrichment analysis

Target and pathway analyses were conducted for significantly differentially expressed miRNAs (FDR ≤0.05) at mid-AAE, post-AAE, and 75 min post-AAE. Using DIANA-microT, we identified 5,336 unique predicted targets at mid-AAE, 7,158 targets at post-AAE, and 1,144 targets at 75 min post-AAE (Supplementary Figure 5). Using PANTHER, we identified enriched Gene Ontology biological processes among the predicted targets at each time point (Bonferroni-adjusted p < 0.05). A total of 231 processes were enriched at mid-AAE, 330 at post-AAE, and 34 at 75 min post-AAE. The ten most enriched processes (ranked by fold enrichment) at each time point are shown in Figure 5A, and the umbrella-category composition of the top 20 enriched processes is summarized in Figure 5B (Supplementary Table 2).

**FIGURE 5:**
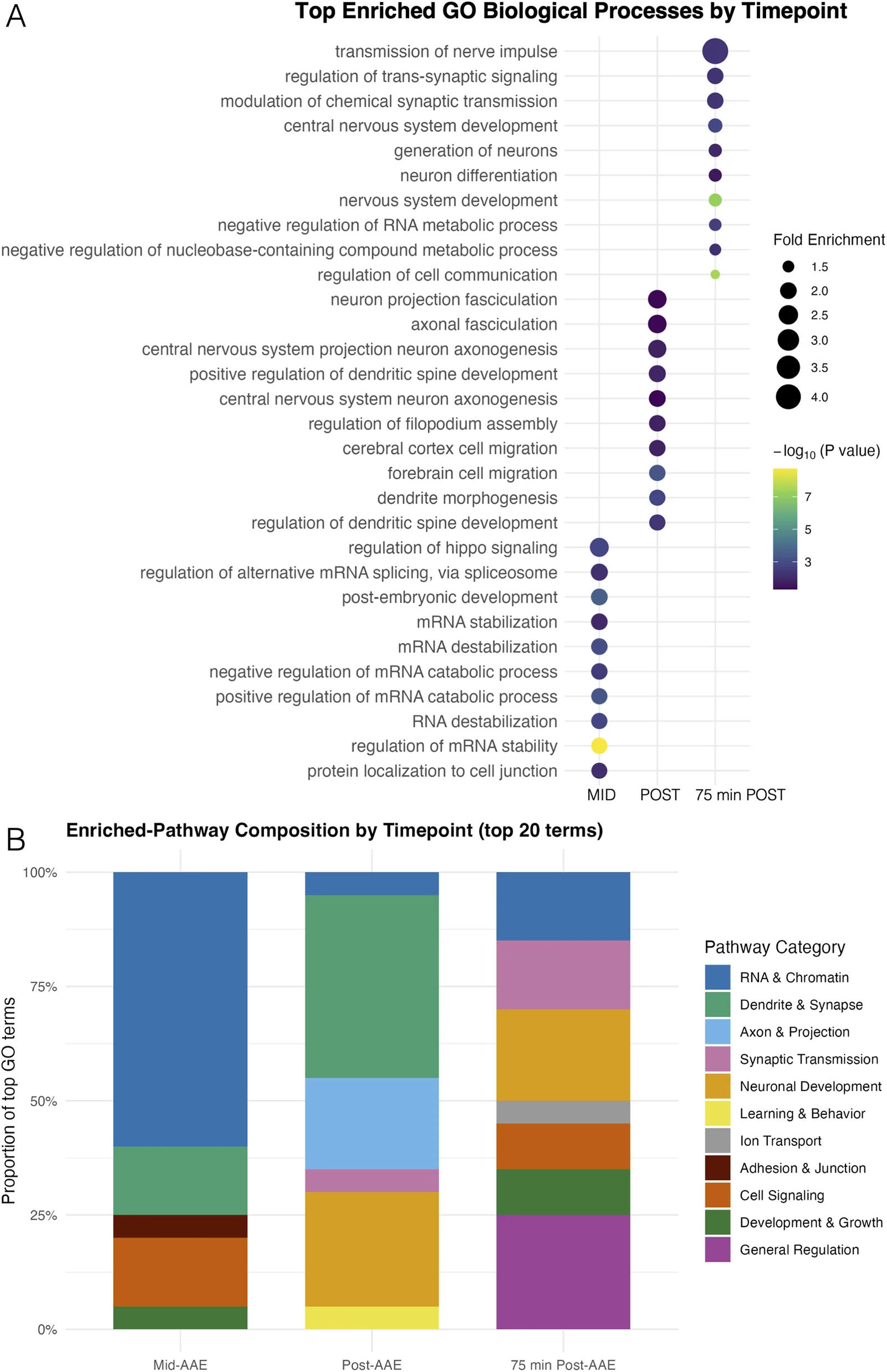
Target mRNA pathway enrichment analysis. **(A)** Bubble plot of the top ten enriched Gene Ontology biological processes among the predicted targets of significantly differentially expressed (FDR ≤0.05) miRNAs at mid-, post-, and 75 min post-AAE. The top ten pathways are a subset of the top 20 pathways, ordered by fold enrichment (highest to lowest). GO terms are ordered on the y-axis by time point and significance, with the most significant at the top. Bubble size represents fold enrichment and color represents the -log10(P value). **(B)** Stacked bar plot showing the umbrella-category composition of the top 20 enriched GO terms at each time point, expressed as the proportion of top GO terms assigned to each category. Categories include RNA & chromatin, dendrite and synapse, axon and projection, synaptic transmission, neuronal development, learning and behavior, ion transport, adhesion and junction, cell signaling, development and growth, and general regulation.

Enrichment followed a clear temporal progression. At mid-AAE, predicted targets were most enriched for RNA processing and stability, including regulation of mRNA stability, mRNA destabilization, RNA stabilization, regulation of mRNA catabolic processes, and regulation of alternative mRNA splicing, alongside regulation of hippo signaling. Immediately post-AAE, enrichment shifted markedly toward neuronal structural remodeling, including regulation of dendritic spine development, dendrite morphogenesis, regulation of filopodium assembly, central nervous system neuron axonogenesis, axonal fasciculation, neuron projection fasciculation, and cerebral cortex and forebrain cell migration. By 75 min post-AAE, the most enriched processes centered on synaptic transmission and neuronal development, including transmission of nerve impulse, modulation of chemical synaptic transmission, regulation of trans-synaptic signaling, nervous system development, neuron differentiation, and generation of neurons. Thus, the predicted-target enrichment progressed from post-transcriptional RNA regulation, to dendritic and synaptic structural organization, to synaptic transmission across the exercise time course.

### 3.5 Hsa-miR-1252-3p, a persistently downregulated EV-Enriched miRNA

hsa-miR-1252-3p was the only miRNA differentially expressed across all three post-exercise time points and was downregulated at each, with a stable effect size (log2 fold change = −2.34 at mid-AAE, −2.34 at post-AAE, and −2.20 at 75 min post-AAE). Normalized expression levels of hsa-miR-1252-3p across the four time points are shown in Figure 6A. This consistency, together with its persistence across the time course, identifies hsa-miR-1252-3p as the most temporally robust EV miRNA signal in our dataset and a candidate marker of the AAE response in MDD. To explore its potential regulatory reach, we identified predicted targets of hsa-miR-1252-3p (1,547 total predicted targets) and tested them for functional enrichment. All enriched Gene Ontology biological processes among its predicted targets are shown in Figure 6B. Among them, the most enriched pathways are related to neuronal processes, including dendrite morphogenesis and development. All enriched pathways are provided in Supplementary Table 3. We note that hsa-miR-1252-3p is largely uncharacterized in the published literature, and these target-based annotations are computational predictions; the specific functional consequences of its downregulation therefore remain to be experimentally validated.

**FIGURE 6:**
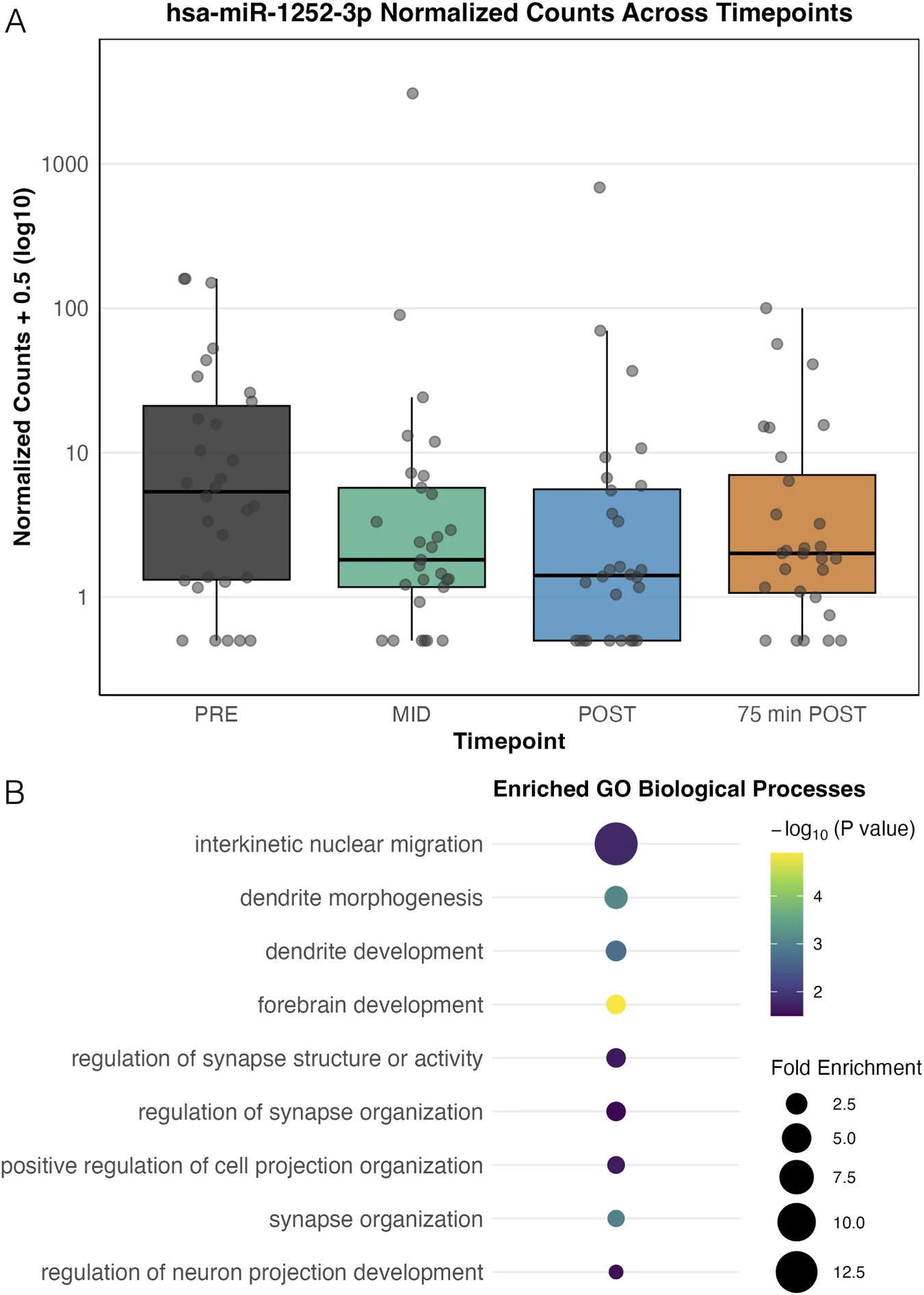
hsa-miR-1252-3p expression and predicted-target enrichment across AAE. **(A)** Normalized counts (log10 of normalized counts +0.5) of hsa-miR-1252-3p at each time point (pre-, mid-, post-, and 75 min post-AAE). Boxes show the median and interquartile range, whiskers extend to 1.5× the interquartile range, and individual points represent samples. **(B)** Bubble plot of the enriched Gene Ontology biological processes among the predicted targets of hsa-miR-1252-3p. Terms are ordered by fold enrichment (most enriched at the top); bubble size represents fold enrichment and color represents the -log10(P value).

To place this signal in a broader tissue context for hypothesis generation, we explored the miRNATissueAtlas (Rishik et al., 2025) for hsa-miR-1252-3p and its opposite-arm strand, hsa-miR-1252-5p. hsa-miR-1252-3p appeared to be more highly expressed in neuronal structures, including the frontal lobe, cortex, grey and white matter, and astrocytes, relative to peripheral tissues such as the lung, liver, heart, and adipose (Supplementary Table 4), although its absolute abundance was low. By contrast, hsa-miR-1252-5p, while also expressed in neuronal structures, showed less neuronal specificity, with comparatively higher expression in peripheral tissues such as heart and liver.

## 4 Discussion

### 4.1 AAE elicits a biphasic and temporally specific response in individuals with MDD

AAE has been shown to improve depressed mood states in individuals with MDD, yet the molecular mechanisms underlying this effect remain incompletely understood. Elucidating the epigenetic effects of aerobic exercise is essential for uncovering how these benefits are mediated. Given their central regulatory roles in biological systems, miRNAs have been proposed as one route through which exercise may exert its effects in MDD. In this study, we hypothesized that AAE would alter the expression of EV-enriched miRNAs with predicted targets involved in pathways relevant to MDD, potentially contributing to its therapeutic benefits. Using next-generation RNA sequencing, we profiled miRNAs in serum EVs of individuals diagnosed with MDD, collected at baseline, during, immediately after, and 75 min following a 30-min bout of aerobic exercise.

Our findings reveal a dynamic and temporally specific miRNA response to AAE. The greatest number of differentially expressed miRNAs occurred immediately post-AAE (33), followed by mid-AAE (15) and 75 min post-AAE (four). The response was also directional: miRNAs were predominantly downregulated at mid- and post-AAE (13 of 15 and all 33, respectively) and exclusively upregulated at the recovery time point (all four). Together with the observation that the differentially expressed miRNAs were largely unique to each time point, this pattern indicates a biphasic, temporally ordered response to exercise rather than a single sustained shift. This dynamic mirrors the canonical circulating-miRNA response to acute exercise reported by Nielsen and colleagues, who found that plasma miRNAs were uniformly downregulated immediately after a bout of aerobic exercise and were subsequently upregulated at one and 3 hours of recovery, with no overlap of differentially expressed miRNAs across time points (Nielsen et al., 2014). A reduction in EV-enriched miRNAs does not reflect fewer circulating EVs; acute exercise generally increases EV release from skeletal muscle and other tissues (Whitham et al., 2018), so one interpretation is that exercise selectively alters which miRNAs are loaded into EVs over time. Consistent with this, the predicted targets of the differentially expressed EV-enriched miRNAs were enriched for distinct biological processes at each time point, as described below.

At mid-AAE, the predicted targets of the downregulated miRNAs were most strongly enriched for post-transcriptional regulatory processes. This signature is consistent with a cell mounting a rapid, stress-responsive adjustment of its post-transcriptional machinery during exercise (Zedan et al., 2025). Because miRNAs repress their targets, a coordinated downregulation of miRNAs whose targets govern RNA stability and translation would be expected to transiently de-repress this machinery, effectively re-tuning gene expression to meet the acute demands of exercise.

Immediately post-AAE, when all 33 differentially expressed miRNAs were downregulated, the enrichment shifted markedly toward neuronal structural organization. Neuronal structural organization is heavily implicated in MDD, with postmortem and preclinical data documenting dendritic spine and synapse loss in the prefrontal cortex and hippocampus (Duman and Aghajanian, 2012). Whether these changes contribute mechanistically to the mood and anhedonia improvements documented in this same population immediately following a single bout of moderate aerobic exercise, or are simply co-occurring, cannot be determined from the associative design; however, the temporal alignment between molecular and behavioral changes makes a mechanistic link a possibility for future causal testing (Meyer et al., 2022).

By 75 min post-AAE, the four differentially expressed miRNAs were upregulated, and their predicted targets were enriched for synaptic transmission and neuronal-development processes. This may represent the functional and plasticity endpoints that aerobic exercise is known to enhance, downstream of the structural remodeling enriched at the immediate post-exercise time point (Li et al., 2020). The reversal of directionality at recovery, from predominant downregulation to exclusive upregulation, is the EV-level counterpart of the circulating-miRNA rebound described by (Nielsen et al., 2014), and may represent a homeostatic, negative-feedback phase that re-establishes baseline gene expression once the acute response has been mounted.

### 4.2 Comparison to previous literature

The observed temporal miRNA predicted target pathway enrichment pattern, including a shift from post-transcriptional regulation, to dendritic and synaptic structural organization, to synaptic transmission, parallels synaptic mechanisms implicated in MDD (Duman et al., 2016; Sanacora et al., 2012), as well as the pro-neuroplastic effects of aerobic exercise (Cotman and Berchtold, 2002). Importantly, exercise-derived EVs, including muscle-derived EVs capable of crossing the blood-brain barrier, have been proposed as a candidate delivery route for such peripheral-to-brain signaling (Khoury and Nagy, 2023; Lerussi et al., 2025; Whitham et al., 2018), a possible mechanism by which AAE may improve symptoms of MDD.

In addition to parallels in enriched pathways, individual differentially expressed miRNAs have previously been implicated in depression, exercise, and synaptic plasticity.

The most strongly supported is hsa-miR-21-5p, which was downregulated immediately post-AAE and is relevant as a depression-associated, EV-packaged, and exercise-responsive miRNA. Serum exosomal miR-21-5p has been reported to track antidepressant treatment response, with lower pre-treatment levels in patients who later achieved remission and increases following treatment (Hung et al., 2021). Baseline miR-21-5p, together with miR-146a-5p, has also been associated with antidepressant efficacy. hsa-miR-183-5p, also downregulated post-AAE, is a member of the miR-183/96/182 cluster (Zhou et al., 2021), which has established links to mTOR-mediated AMPA-receptor regulation (Xie et al., 2017). hsa-miR-224-5p, downregulated at both mid- and post-AAE, is biologically notable because it is intronic to and co-transcribed with GABRE, the gene encoding the epsilon subunit of the GABA-A receptor, providing a direct genomic tie to inhibitory neurotransmission, and its inhibition restrains neuronal apoptosis *via* NR4A1 (Liu et al., 2020). Finally, hsa-miR-92a-1-5p, downregulated post-AAE, is a regulator of homeostatic synaptic plasticity: miR-92a represses translation of GluA1-containing AMPA receptors, and its activity-dependent reduction de-represses local dendritic GluA1 during synaptic scaling (Letellier et al., 2014), reinforcing the dendrite-and-synapse theme of our enrichment results.

The remaining differentially expressed miRNAs, including the recovery-phase differentially expressed miRNAs and the majority of the mid- and post-AAE sets, have little or no established neuro-, depression-, or exercise-related literature and are best regarded as novel, largely uncharacterized signals in this context. Overall, the miRNAs with the clearest prior support converge on synaptic and neurotrophic mechanisms, consistent with exercise engaging the same plasticity pathways implicated in MDD.

### 4.3 miR-1252-3p as a novel, persistently downregulated miRNA in AAE

The single most distinctive signal in our dataset was hsa-miR-1252-3p, the only miRNA differentially expressed across all three post-exercise time points, downregulated at each with a stable effect size (log2 fold change ≈ −2.3).

hsa-miR-1252-3p is an essentially uncharacterized microRNA. It derives from the hsa-mir-1252 hairpin (miRBase accession MI0006434; mature 3p product MIMAT0026744) at chromosome 12q21.2, and there are no primary studies that experimentally characterize the 3p strand, with no validated targets and no documented role in brain, synapse, depression, exercise, or extracellular-vesicle biology (Kozomara et al., 2019). The only substantive functional data for the locus belong to the opposite arm, miR-1252-5p, a tumor-suppressive, EV-associated strand reported to target heparinase (Rodrigues- Junior et al., 2021); because of strand specificity, that literature cannot be assumed to apply to the 3p product detected here.

Given this limited characterization, the present data offer a starting point for further investigation. Its brain-enriched tissue distribution, together with the synaptic functional enrichment of its predicted targets, reinforces hsa-miR-1252-3p as a candidate for further characterization in the brain. Investigating its specific cellular targets and regulatory role in the context of exercise and MDD may provide insight into the molecular mechanisms underlying exercise-induced neuroplasticity. Notably, its expression in muscle tissue remains poorly characterized, warranting further investigation into the mechanisms by which AAE may regulate its levels in this context. Future studies should characterize the surface markers of EVs released during AAE to determine their tissue of origin and potential target destinations.

We stress that these annotations are computational predictions and that any specific synaptic or depression-related role for hsa-miR-1252-3p remains speculative. As such, the consistent suppression of hsa-miR-1252-3p across the exercise time course is best presented as a candidate marker of the AAE response in MDD and a priority for targeted experimental follow-up.

### 4.4 Strengths and contributions

There are several strengths to this written work. We used a non-invasive procedure to identify key miRNAs in individuals diagnosed with MDD, in relation to aerobic exercise, by collecting serum samples. miRNAs were profiled from an EV-enriched serum fraction isolated by membrane-affinity chromatography, which is expected to be enriched for vesicle-associated miRNAs relative to total serum and to reduce contamination from cell-free or protein-bound RNA species. Additionally, we have implemented comprehensive profiling of miRNAs using RNA-seq, which should reflect all miRNAs in our system, including low-abundance ones. Furthermore, this study included several timepoints, including pre-, mid-, post-, and 75 min post-AAE, allowing for a more comprehensive understanding of the transient and delayed effects of AAE on miRNA expression of individuals diagnosed with MDD. This led to the discovery of a time-dependent, biphasic effect of AAE on serum EV-enriched miRNAs. Furthermore, to our knowledge, this study is the first to examine how AAE affects the expression of serum EV-enriched miRNAs in individuals with MDD, thereby bridging the gap between behavioral outcomes and molecular mechanisms.

### 4.5 Limitations

Several considerations should inform interpretation of these findings. Neuronal-derived EVs were not isolated using neuronal surface markers; therefore, the profiled miRNAs represent the total serum EV pool rather than a brain- or neuron-specific subpopulation. The tissue origin of the differentially expressed miRNAs cannot be determined, and neurobiological interpretations should be considered preliminary. Future studies using neuronal-marker-based enrichment or complementary tissue-of-origin approaches will help determine whether these responses reflect central nervous system signaling. In addition, because the exoEasy method can co-isolate non-vesicular RNA carriers, including lipoproteins, the analyzed fraction is best described as EV-enriched rather than exclusively vesicular.

Preanalytical factors may also have influenced the serum EV-enriched miRNA profile. Hemolysis, assessed quantitatively *post hoc*, did not differ significantly across timepoints. Serum use, clotting time, and exercise-induced platelet activation may have increased platelet-derived EV representation; miR-21-5p and miR-92a-1-5p showed greater variability across timepoints than other platelet markers tested, though this did not reach significance after correction, and findings for these two miRNAs should be interpreted with caution.

The sample was predominantly female, with 21 of 30 participants identifying as female, and was not sufficiently powered to evaluate sex-specific responses. Although this distribution reflects the parent trial, larger, sex-balanced cohorts are needed to determine whether the observed biphasic EV-enriched miRNA pattern generalizes across sexes. The sample size was appropriate for an exploratory analysis, but replication in larger and more clinically diverse cohorts will be important to confirm these findings and assess their relevance to MDD subtypes, including treatment-resistant depression.

The present study lacked both a healthy comparison group and EV-enriched miRNA data from the parent study's seated-rest condition. Consequently, it remains unclear whether the observed EV-enriched miRNA response is specific to individuals with MDD or differs from that of adults without MDD. Similarly, without a time-matched resting comparator, temporal changes cannot be attributed exclusively to exercise rather than to repeated venipuncture, anticipatory stress, circadian variation, or other time-dependent factors. Future studies incorporating both comparison groups will help distinguish exercise-specific effects from MDD-associated responses.

Finally, the mechanistic interpretation of this sequencing-based analysis is limited by the availability of experimentally validated miRNA targets, particularly for hsa-miR-1252-3p. The functional implications of these findings are therefore hypothesis-generating and will require experimental validation. Nonetheless, this study provides foundational evidence of the EV-enriched miRNA response to acute aerobic exercise in individuals with MDD, identifies candidate miRNAs for further investigation, and establishes a framework for future mechanistic and translational studies.

### 4.6 Future directions

The most immediate priority is to functionally characterize hsa-miR-1252-3p, the persistently downregulated and largely uncharacterized miRNA identified here. As a first step, predicted targets generated for the 3p strand (MIMAT0026744) should be experimentally validated using luciferase reporter assays to confirm direct targeting. Prioritizing predicted targets that fall within the dendrite-, synapse-, and neuron-projection pathways enriched in our analysis would directly test whether miR-1252-3p regulates the neuronal programs implicated in MDD.

Beyond target validation, it will be important to determine how brain regions affected in MDD respond to a differentially expressed miRNA at the cellular level. Neuronal cultures derived from or modeling MDD-relevant regions, such as the prefrontal cortex and hippocampus, could be exposed to miR-1252-3p mimics or inhibitors (or to the corresponding EVs), with readouts including dendritic spine density and morphology, synaptic marker expression, and de-repression of target genes. This *in vitro* approach would determine whether modulating a single exercise-responsive EV-enriched miRNA is sufficient to produce the molecular and structural changes predicted by our pathway analysis and would help distinguish cell-autonomous effects from those requiring EV-mediated delivery.

Finally, *in vivo* work could test the behavioral relevance of these EV-enriched miRNAs using an acute-stress rodent model. Across all these directions, correlating EV-enriched miRNA shifts with clinical measures and peripheral BDNF, isolating neuron- and muscle-derived EV subpopulations, and replicating findings in larger, sex-balanced cohorts will be essential.

### 4.7 Concluding remarks

Our findings highlight temporally specific changes co-occurring with acute aerobic exercise in individuals diagnosed with MDD, with possible activation of dendritic plasticity through predicted targets, most prominent at 75 min post-exercise. These results identify candidate miRNAs relevant to how exercise may influence brain function in depression, without establishing a causal or mechanistic role. Results provide a framework for future hypothesis-driven research, particularly regarding which miRNAs should be prioritized for functional validation in the context of AAE and MDD. Our findings inform the design of future interventions and broaden the conceptual framework for how lifestyle modifications like exercise can influence the underlying biology of depressive disorders.

## Data Availability

The datasets presented in this study can be found in online repositories. The names of the repository/repositories and accession number(s) can be found below: https://www.ncbi.nlm.nih.gov/geo/, GSE320360.

## Footnotes

1
https://www.bioinformatics.babraham.ac.uk/projects/fastqc/ (Accessed 22 September 2025).

2
https://sourceforge.net/projects/bbmap/(Accessed 22 September 2025).
