# Supplementary material for: Acute aerobic exercise is associated with a biphasic, temporally ordered shift in serum extracellular vesicle microRNA expression in major depressive disorder
Source: Front Mol Biosci. 2026 Sep 15;13:1910088. doi: 10.3389/fmolb.2026.1910088 (PMC13619381; doi:10.3389/fmolb.2026.1910088)

Supplementary Material

1 Supplementary Tables

Supplementary Table 1. Vesicle size distribution and reproducibility by sample and replicate. Median diameter, geometric mean, coefficient of variation, standard deviation, and 5th/95th percentiles calculated per sample and replicate from calibrated vFRed fluorescence intensity on the ImageStream.

Sample	Replicate	Events	Median (nm)	Geo. Mean (nm)	CV (%)	SD (nm)	5th %ile	95th %ile
**E1**	R1	33016	103.20	106.20	11.00	11.74	95.92	126.87
**E1**	R2	28553	103.78	106.50	11.01	11.78	98.37	126.96
**E2**	R1	16086	104.94	108.84	14.48	15.89	96.11	137.34
**E2**	R2	14930	104.94	109.00	14.48	16.47	95.69	140.59
**E3**	R1	34168	105.14	109.67	16.07	17.81	95.73	143.83
**E3**	R2	27299	105.39	110.07	16.15	17.96	95.78	145.41
**E6**	R1	25848	107.11	112.30	17.68	20.11	96.33	151.88
**E6**	R2	26902	107.13	112.48	17.88	20.37	96.49	153.22

Supplementary Table 2. Gene Ontology enrichment of predicted miRNA targets. Predicted mRNA targets of differentially expressed miRNAs were identified using DIANA-microT 2023 (score ≥0.8) and functionally annotated using PANTHER GO Biological Processes. Columns show: Term, number of genes, fold enrichment, and Bonferroni-adjusted p-value. Results are stratified by time point and ranked by fold enrichment. Only the top 20 pathways, based on fold enrichment, are shown.

**Mid-AAE vs Pre-AAE**			
**Term**	**Genes**	**FoldEnrichment**	**Pvalue**
**regulation of hippo signaling**	29	2.4	0.00159
**regulation of alternative mRNA splicing, via spliceosome**	38	2.07	0.00701
**post-embryonic development**	51	2.02	2.81E-04
**mRNA stabilization**	40	2	0.0108
**mRNA destabilization**	49	1.98	9.14E-04
**negative regulation of mRNA catabolic process**	46	1.97	0.00319
**regulation of mRNA stability**	99	1.94	1.89E-09
**RNA destabilization**	50	1.94	0.00175

**positive regulation of mRNA catabolic process**	55	1.94	5.52E-04
**protein localization to cell junction**	47	1.9	0.00783
**regulation of translational initiation**	48	1.9	0.00906
**dendrite development**	57	1.89	8.79E-04
**RNA stabilization**	44	1.88	0.0261
**regulation of RNA stability**	102	1.87	1.66E-08
**regulation of postsynapse organization**	69	1.85	1.23E-04
**negative regulation of RNA catabolic process**	50	1.85	0.013
**regulation of dendrite development**	48	1.85	0.0254
**positive regulation of mRNA metabolic process**	64	1.85	4.08E-04
**negative regulation of transforming growth factor beta receptor signaling pathway**	46	1.84	0.0323
**negative regulation of transmembrane receptor protein serine/threonine kinase signaling pathway**	74	1.84	4.58E-05
**Post-AAE vs Pre-AAE**			
**Term**	**Genes**	**FoldEnrichment**	**Pvalue**
**axonal fasciculation**	19	2.32	0.0473
**neuron projection fasciculation**	19	2.32	0.0473
**central nervous system projection neuron axonogenesis**	23	2.23	0.0174
**positive regulation of dendritic spine development**	30	2.05	0.0129
**central nervous system neuron axonogenesis**	28	2.01	0.0492
**regulation of filopodium assembly**	33	1.97	0.0166
**cerebral cortex cell migration**	34	1.95	0.0158
**forebrain cell migration**	45	1.94	5.60E-04
**regulation of dendritic spine development**	39	1.92	0.00496
**dendrite morphogenesis**	43	1.92	0.00175
**telencephalon cell migration**	42	1.9	0.00316
**regulation of postsynaptic membrane neurotransmitter receptor levels**	47	1.88	9.85E-04
**protein localization to synapse**	43	1.83	0.0157
**associative learning**	62	1.76	6.05E-04
**regulation of postsynapse organization**	88	1.74	1.57E-06
**neuron migration**	90	1.73	1.53E-06
**regulation of synapse structure or activity**	201	1.72	1.26E-17
**central nervous system neuron development**	59	1.72	0.00266
**positive regulation of mRNA catabolic process**	66	1.71	7.72E-04
**regulation of synapse organization**	196	1.71	1.11E-16
**75 min Post-AAE vs Pre-AAE**			
**Term**	**Genes**	**FoldEnrichment**	**Pvalue**

**transmission of nerve impulse**	16	4.3	0.00486
**modulation of chemical synaptic transmission**	60	1.97	0.00502
**regulation of trans-synaptic signaling**	60	1.97	0.00518
**central nervous system development**	101	1.69	0.00146
**generation of neurons**	111	1.59	0.011
**nervous system development**	209	1.58	6.68E-08
**neuron differentiation**	104	1.58	0.0271
**negative regulation of RNA metabolic process**	137	1.54	0.00256
**negative regulation of nucleobase-containing compound metabolic process**	144	1.5	0.00682
**regulation of cell communication**	305	1.44	3.51E-08
**regulation of signaling**	303	1.43	7.80E-08
**system development**	303	1.43	1.49E-07
**multicellular organism development**	333	1.41	2.35E-08
**regulation of signal transduction**	254	1.37	5.82E-04
**regulation of biological quality**	242	1.36	0.00278
**negative regulation of macromolecule metabolic process**	208	1.36	0.031
**positive regulation of macromolecule metabolic process**	245	1.32	0.0189
**negative regulation of cellular process**	400	1.31	2.56E-06
**regulation of RNA metabolic process**	277	1.29	0.0287
**negative regulation of biological process**	409	1.28	3.16E-05

Supplementary Table 3. Gene Ontology enrichment of predicted hsa-miR-1252-3p targets. Predicted mRNA targets of differentially expressed miRNAs were identified using DIANA-microT 2023 (score ≥0.8) and functionally annotated using PANTHER GO Biological Processes. Columns show: Term, number of genes, fold enrichment, and Bonferroni-adjusted p-value. Results are stratified by time point and ranked by fold enrichment.

Term	Genes	FoldEnrichment	Pvalue
**interkinetic nuclear migration**	5	13.41	0.016
**dendrite morphogenesis**	30	2.87	0.00083
**dendrite development**	41	2.34	0.002
**forebrain development**	66	2.17	0.000013
**regulation of synapse structure or activity**	40	2.15	0.024
**regulation of synapse organization**	39	2.15	0.032
**positive regulation of cell projection organization**	52	1.94	0.023
**synapse organization**	70	1.9	0.00095
**regulation of neuron projection development**	62	1.81	0.029

Supplementary Table 4. Tissue expression of hsa-miR-1252-3p and hsa-miR-1252-5p from the miRNATissueAtlas. Mean expression (reads per million, RPM) and standard deviation (SD) of hsa-miR-1252-3p and hsa-miR-1252-5p across peripheral tissues, whole brain, and brain subregions, based on the miRNATissueAtlas (Rishik et al., 2025).

Tissue / Region	hsa-miR-1252-3p Mean RPM (SD)	hsa-miR-1252-5p Mean RPM (SD)
Peripheral tissues		
**Adipose**	0.0000 (0.0000)	0.0498 (0.0359)
**Lung**	0.0133 (0.1867)	0.1045 (1.2044)
**Liver**	0.0338 (0.1301)	1.2910 (2.3241)
**Heart**	0.0572 (0.1645)	2.5201 (2.9190)
**Nerve**	0.0585 (0.0750)	0.2145 (0.1334)
Brain (organ level)		
**Brain**	0.3469 (1.6105)	2.3212 (4.5674)
Brain subregions		
**Cortex**	0.2138 (0.2993)	1.0932 (1.0904)
**Grey matter**	0.2171 (0.4000)	1.7227 (2.6458)
**White matter**	0.2510 (0.1838)	2.1650 (1.7403)
**Astrocytes**	0.6708 (3.0111)	0.9488 (4.7402)
**Frontal lobe**	0.9521 (0.3907)	3.5720 (0.6233)

2 Supplementary Figures

Supplementary Figure 1. Quality control and exploratory analysis of small RNA-seq data. (a) Barplot of library sizes after variance-stabilizing transformation (VST), which reduces heteroscedasticity and facilitates sample comparison. (b) Principal component analysis (PCA) plot of VST-normalized counts, depicting sample clustering across the first two principal components. Each point represents a sample, colored by group or batch as indicated in the legend. (c) Cook's distance (log10) for each sample, highlighting the influence of each sample on the fitted model; larger values indicate greater influence. (d) Dispersion estimates for each miRNA as a function of mean normalized counts. The fitted dispersion trend is shown in red, with individual gene estimates in black and final values in blue. (e) Barplot of the top 20 most highly expressed miRNAs across all samples, based on mean VST-normalized expression values.

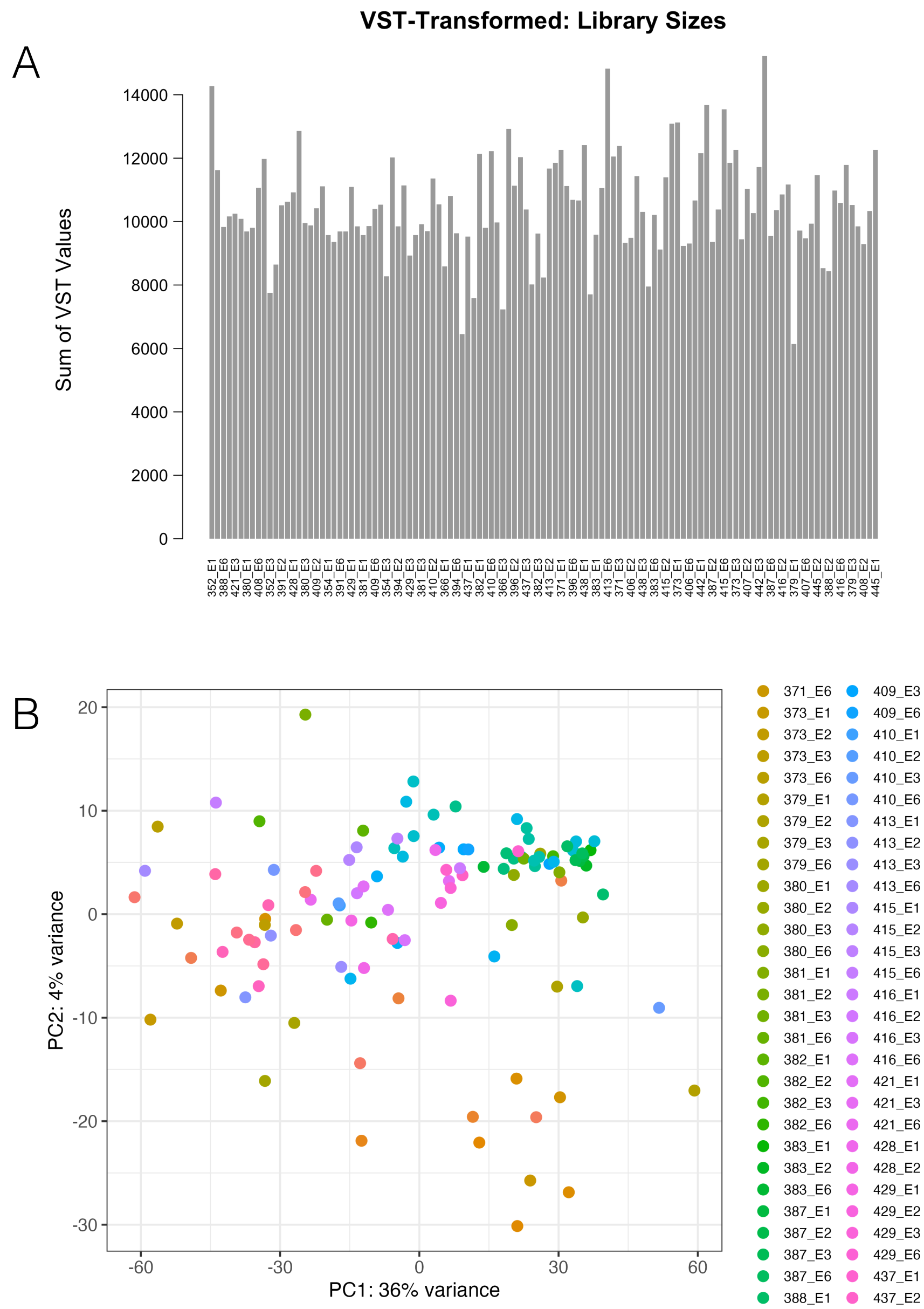

C

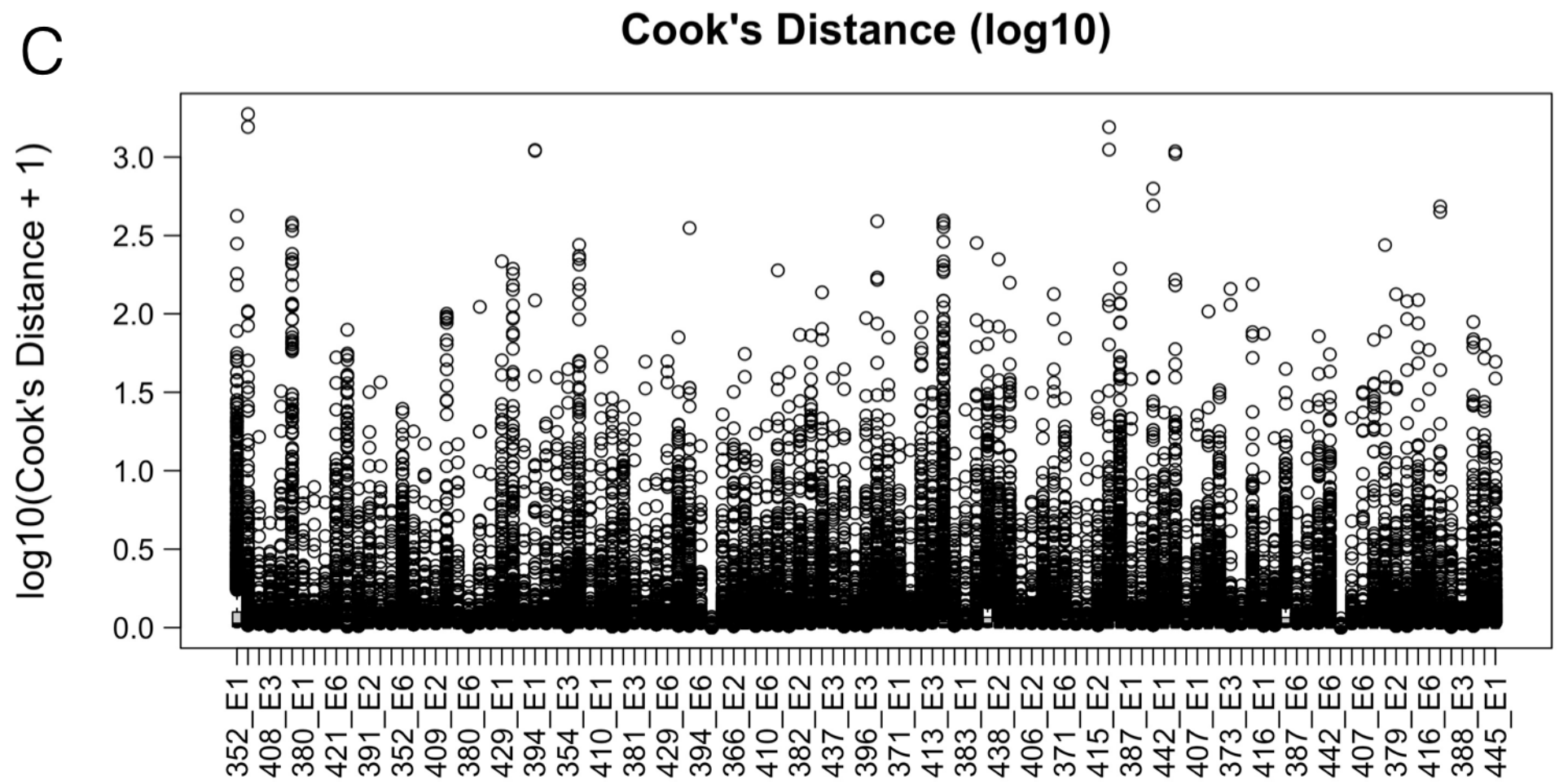
Cook's Distance (log10)
log10(Cook's Distance + 1)
0.0
0.5
1.0
1.5
2.0
2.5
3.0
352_E1
408_E3
380_E1
421_E6
391_E2
352_E6
409_E2
380_E6
429_E1
394_E1
354_E3
410_E1
381_E3
429_E6
394_E6
366_E2
410_E6
382_E2
437_E3
396_E3
371_E1
413_E3
383_E1
438_E2
406_E2
371_E6
415_E2
387_E1
442_E1
407_E1
373_E3
416_E1
387_E6
442_E6
407_E6
379_E2
416_E6
388_E3
445_E1

D

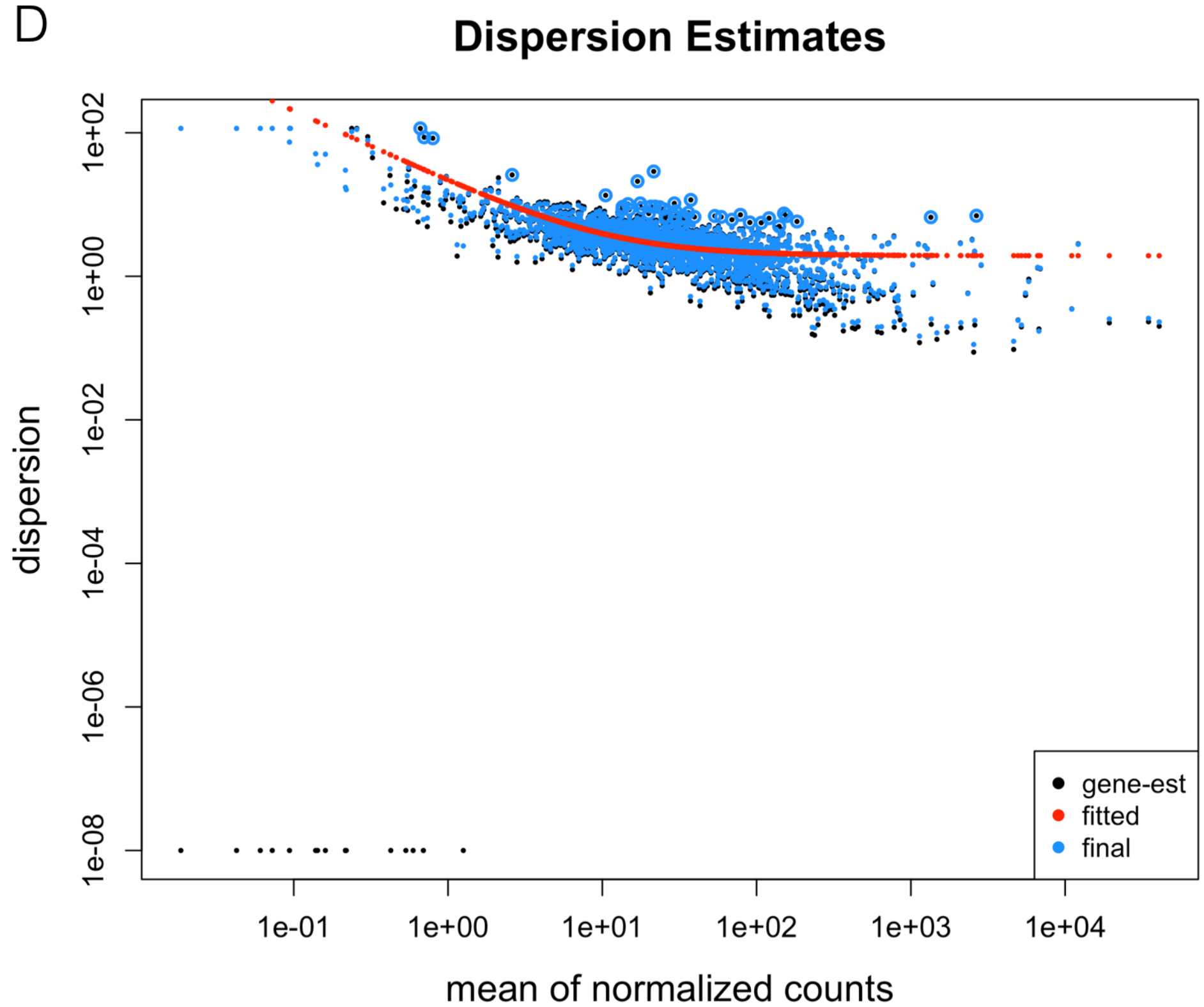
Dispersion Estimates
dispersion
1e-08
1e-06
1e-04
1e-02
1e+00
1e+02
1e-01
1e+00
1e+01
1e+02
1e+03
1e+04
mean of normalized counts
gene-est
fitted
final

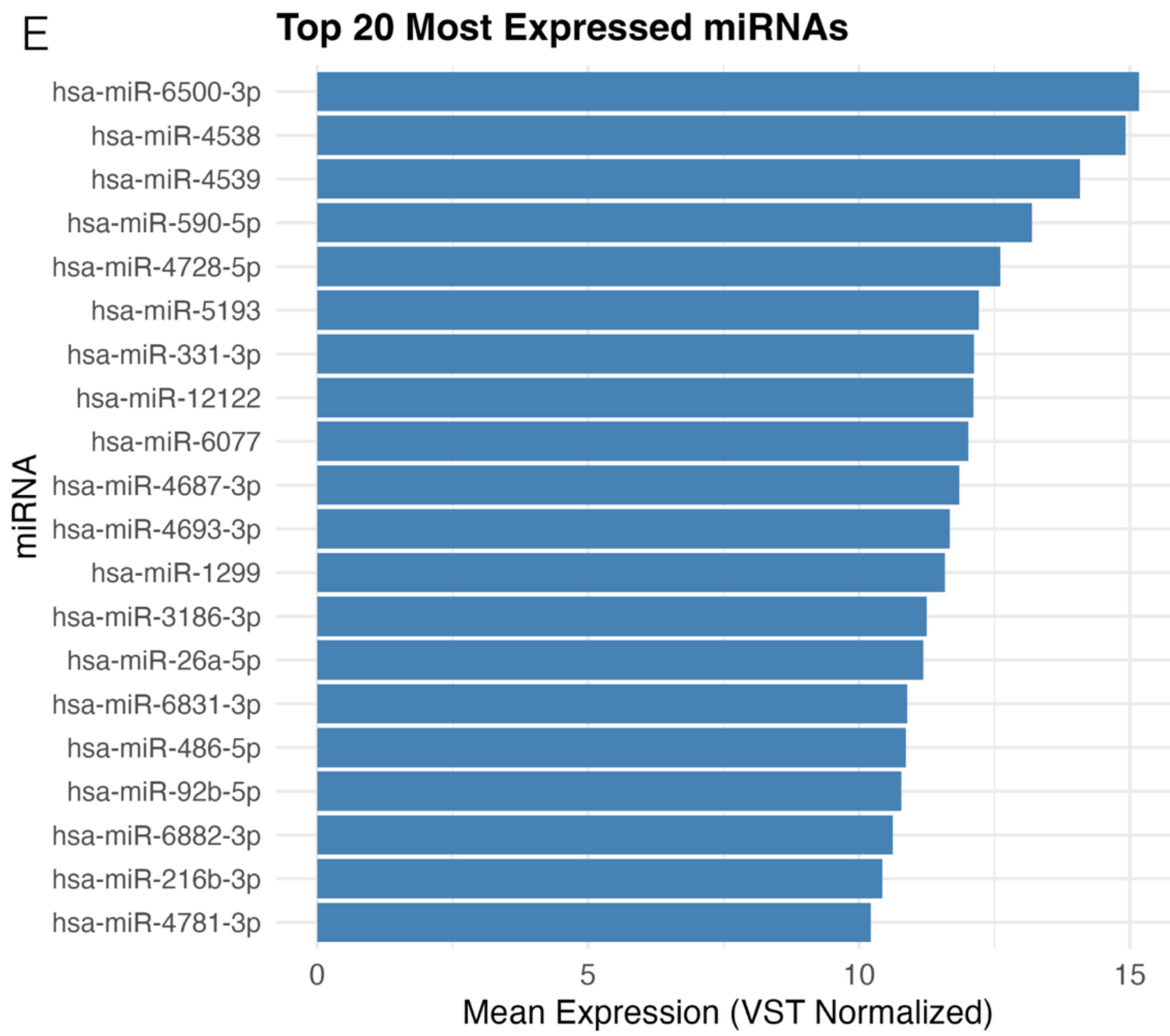
E
Top 20 Most Expressed miRNAs
miRNA
hsa-miR-6500-3p
hsa-miR-4538
hsa-miR-4539
hsa-miR-590-5p
hsa-miR-4728-5p
hsa-miR-5193
hsa-miR-331-3p
hsa-miR-12122
hsa-miR-6077
hsa-miR-4687-3p
hsa-miR-4693-3p
hsa-miR-1299
hsa-miR-3186-3p
hsa-miR-26a-5p
hsa-miR-6831-3p
hsa-miR-486-5p
hsa-miR-92b-5p
hsa-miR-6882-3p
hsa-miR-216b-3p
hsa-miR-4781-3p
0
5
10
15
Mean Expression (VST Normalized)

Supplementary Figure 2. Post-hoc hemolysis assessment. Upper panel: strip/boxplot of the hemolysis index (log2 miR-451a:miR-23a-3p ratio) for each sample, grouped by exercise timepoint. Individual points represent single samples; the horizontal dashed line marks the conventional hemolysis threshold (log2 ratio = 2.81), above which samples are flagged as potentially hemolyzed. Hemolysis index values were scattered across a wide range with no consistent shift in distribution across timepoints, consistent with the Friedman test result (p = 0.472) indicating no systematic hemolysis effect of the exercise intervention. Lower panel: principal component analysis (PCA) of variance-stabilized miRNA expression across all samples, colored by hemolysis index. No discernible clustering by hemolysis index was observed along PC1 (36% variance) or PC2 (4% variance), supporting the conclusion that hemolysis did not meaningfully influence global miRNA expression patterns.

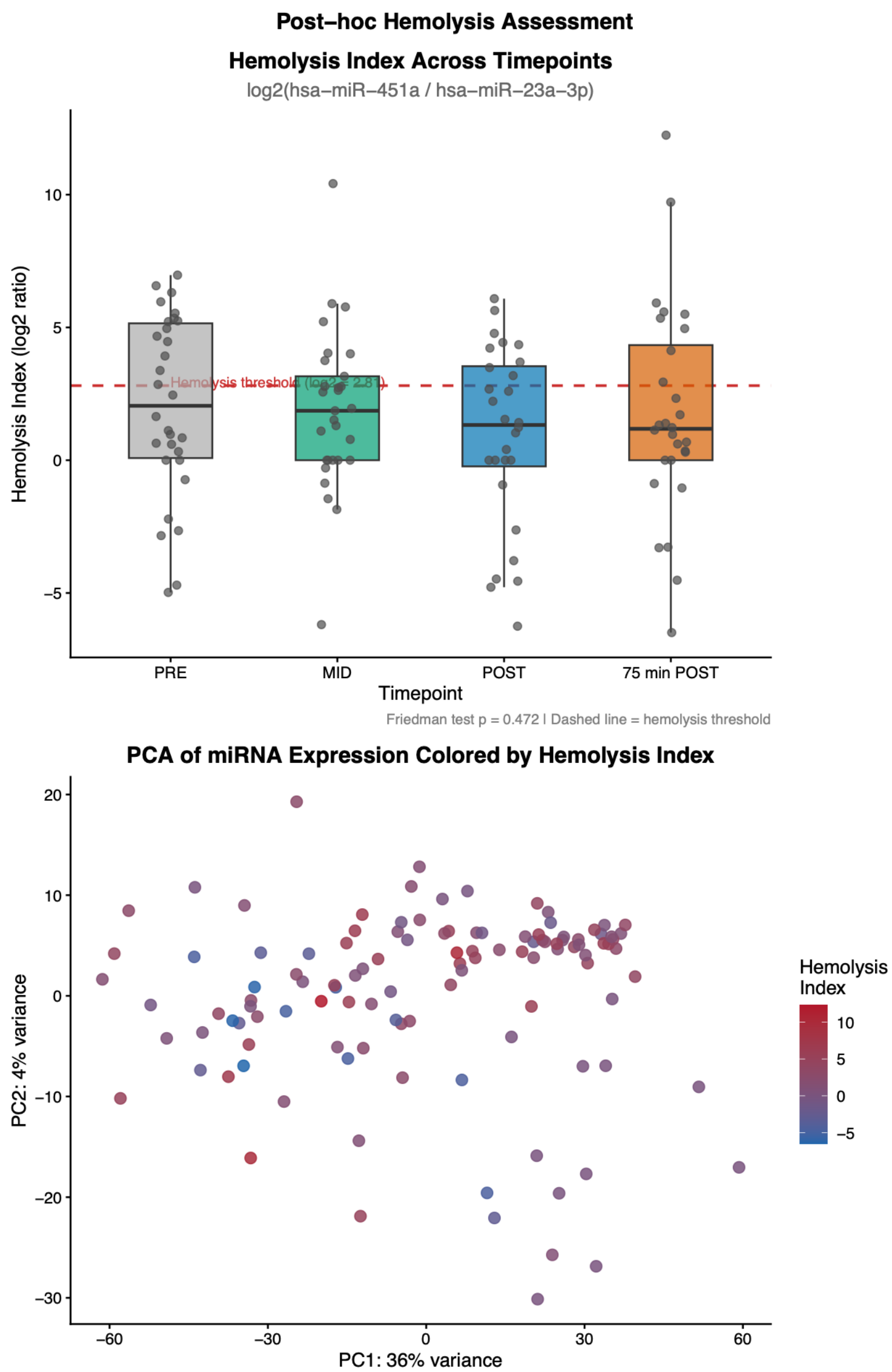

Supplementary Figure 3. Post-hoc platelet contamination assessment. Boxplots showing normalized expression (log2 + 1) of five platelet-enriched miRNAs (miR-223-3p, miR-126-3p, miR-21-5p, miR-92a-1-5p, and miR-191-5p) for each sample, grouped by exercise timepoint and faceted by miRNA. Points are colored by whether the miRNA was also identified as differentially expressed in the primary analysis. Friedman tests ($n = 26$ subjects; Benjamini-Hochberg-adjusted across the five miRNAs) showed no significant timepoint-associated change for any platelet miRNA (all p-adj $\geq$ 0.167); miR-21-5p reached nominal significance before correction ($p = 0.049$) and, along with miR-92a-1-5p, showed visibly greater variability across timepoints than the other three miRNAs tested.

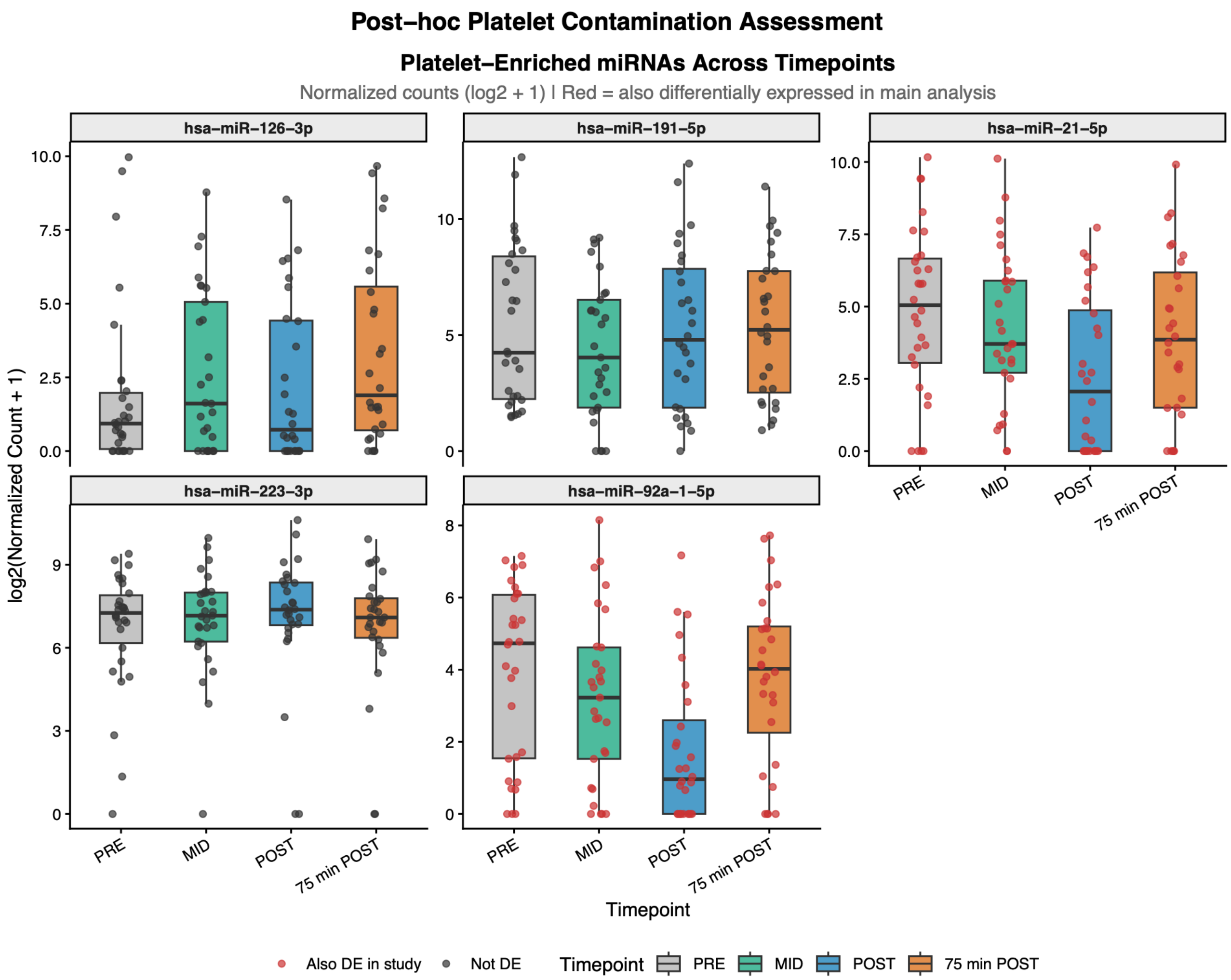

Supplementary Figure 4. PCA of serum EV miRNA expression across exercise timepoints. Each point represents an individual sample, colored by time point. Confidence ellipses (95%) show clustering by group. Separation between timepoints indicates distinct miRNA profiles at each exercise stage.

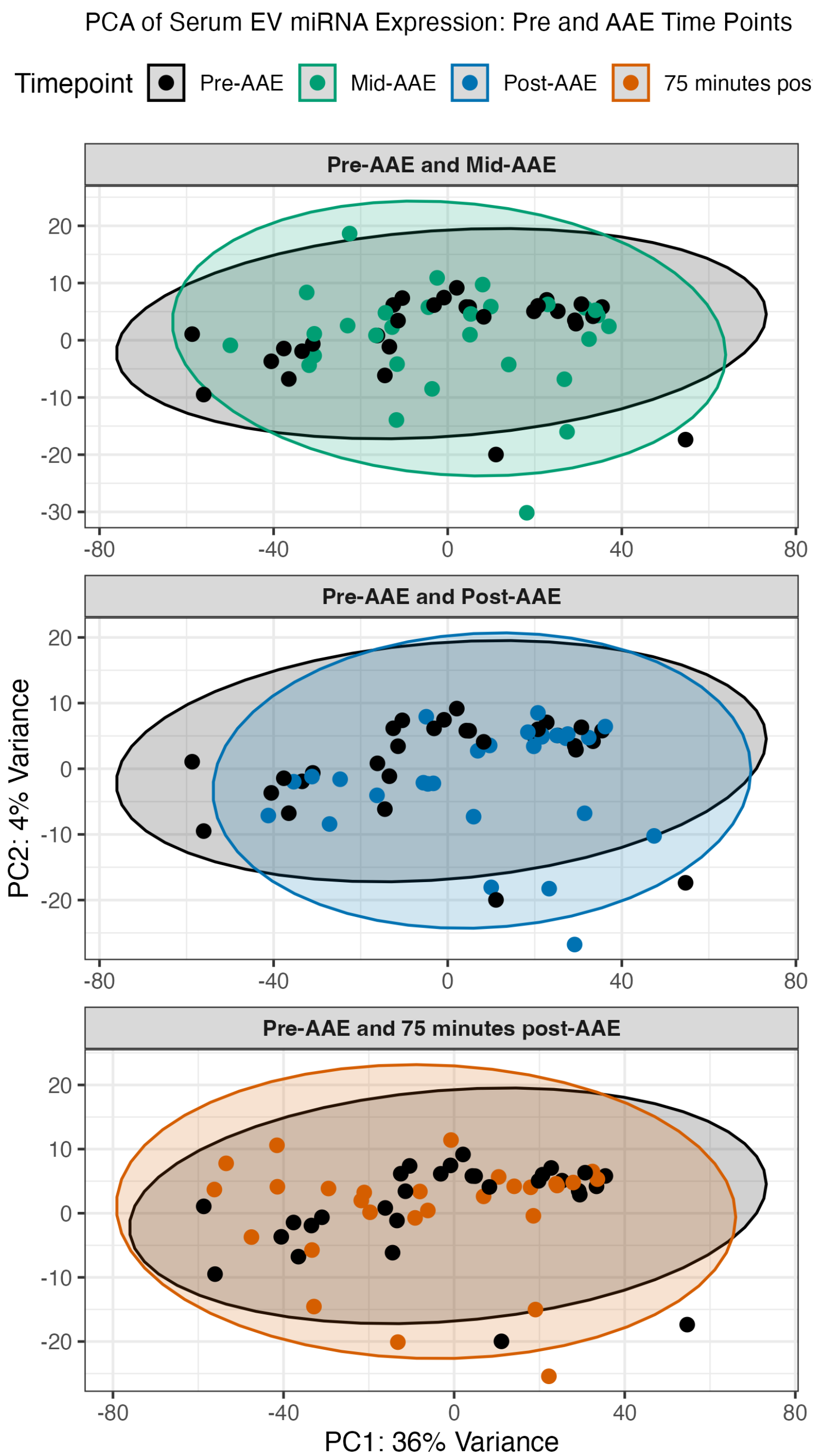

Supplementary Figure 5. miRNA target prediction. Venn diagram of total mRNA targets in each time point, and the number of shared predictions across time.

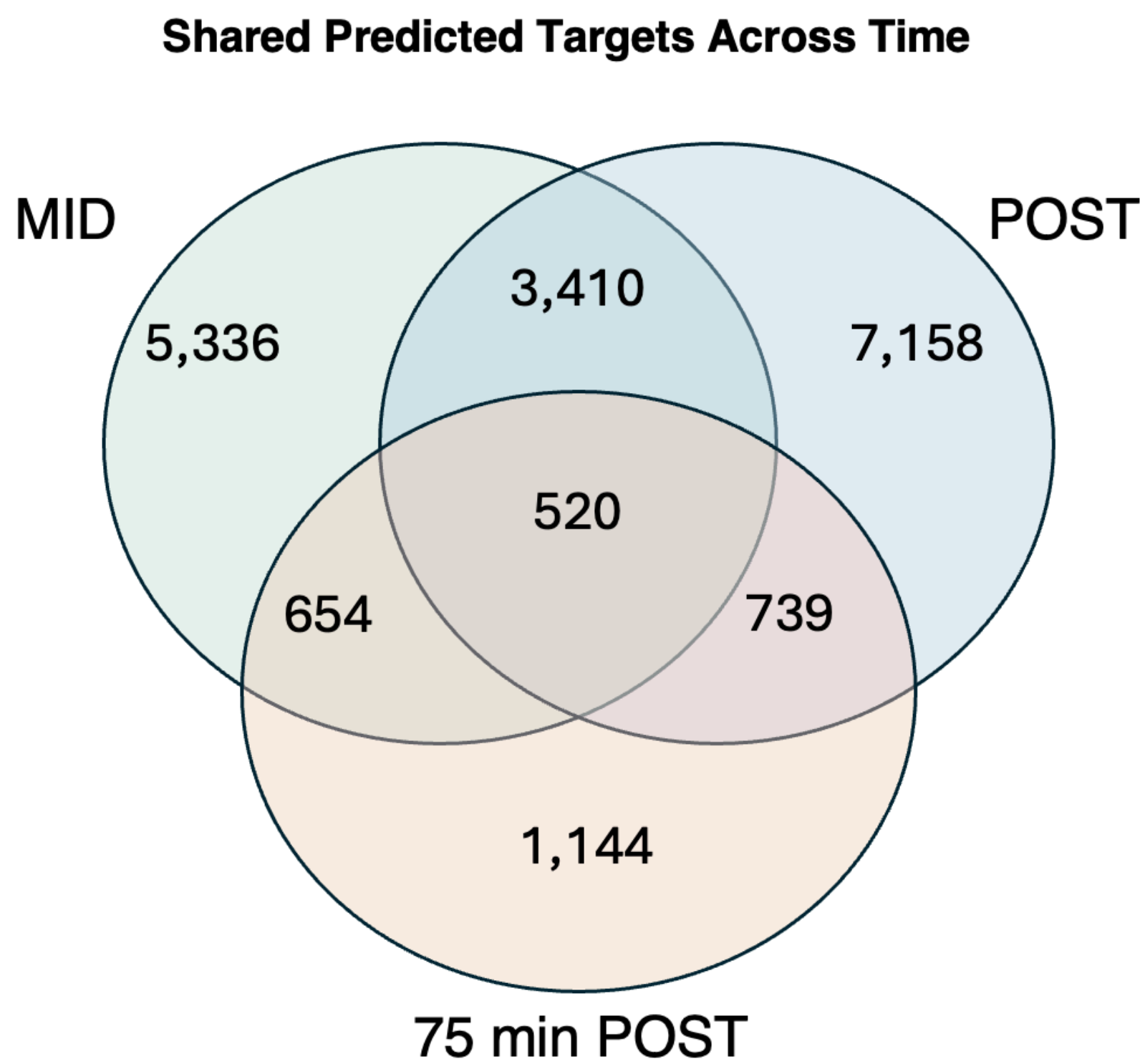

Supplement: Supplementary file 1 [file DataSheet1.pdf]
